# Self-assembly of 2,3-dihydroxycholestane steroids into supramolecular organogels as a soft template for the in-situ generation of silicate nanomaterials

**DOI:** 10.3762/bjoc.9.213

**Published:** 2013-09-09

**Authors:** Valeria C Edelsztein, Andrea S Mac Cormack, Matías Ciarlantini, Pablo H Di Chenna

**Affiliations:** 1 Departamento de Química Orgánica and UMYMFOR (CONICET-FCEN), Facultad de Ciencias Exactas y Naturales, Universidad de Buenos Aires, Ciudad Universitaria, Pabellón II, Buenos Aires, C1428EGA, Argentina

**Keywords:** Hansen parameters, nanotube, organogel, self-assembly, supramolecular gel

## Abstract

Supramolecular gels are an important and interesting class of soft materials that show great potential for many applications. Most of them have been discovered serendipitously, and understanding the supramolecular self-assembly that leads to the formation of the gel superstructure is the key to the directed design of new organogels. We report herein the organogelating property of four stereoisomers of the simple steroid 2,3-dihydroxycholestane. Only the isomer with the *trans*-diaxial hydroxy groups had the ability to gelate a broad variety of liquids and, thus, to be a super-organogelator for hydrocarbons. The scope of solvent gelation was analysed with regard to two solvent parameters, namely the Kamlet–Taft and the Hansen solubility parameters. The best correlation was observed with the Hansen approach that revealed the existence of two clear gelation zones. We propose a general model of self-assembly through multiple intermolecular hydrogen bonds between the 1,2-dihydroxy system, which is based on experimental data and computational simulations revealing the importance of the di-axial orientation of the hydroxy groups for the one-dimensional self-assembly. Under controlled conditions, the fibrillar superstructure of the organogel was successfully used as a template for the in-situ sol–gel polymerization of tetraethoxysilane and the further preparation of silica nanotubes. We propose that the driving forces for templating are hydrogen bonding and electrostatic interactions between the anionic silicate intermediate species and the self-assembled fibrillar network.

## Introduction

Low molecular mass organogelators (LMOGs) have received increasing attention during the last two decades because of their unique properties and numerous potential applications in fields such as the stabilization of organic photochromatic materials, the templated synthesis of nanostructured and functional materials, the controlled release drugs systems, the capture of spilled pollutants in the environment, electrochemistry, light-harvesting materials and so on [1–6]. These small molecules self-assemble into regular supramolecular structures through non covalent interactions such as ion–ion, dipole–dipole, hydrogen bonding, π–π stacking, van der Waals, host–guest, and ion coordination, and in so doing trap the solvent molecules in the supramolecular network to form supramolecular gels. The non-covalent nature of these interactions makes it possible for the supramolecular gel systems to achieve a reversible sol–gel phase transition by the simple application of an external stimulus. Intrinsically, supramolecular gels are thermosensitive and can be transformed reversibly to a fluid (sol) by heating. A small number of novel LMOGs, however, undergo a sol–gel transition as the temperature increases, which is called thermogelling [7]. Many other LMOG molecules form gels that are sensitive to other physical stimuli such as light, ultrasound or chemical stimuli [8–12]. A wide variety of structurally diverse molecules have the ability to form physical gels (e.g., saccharides, peptides, ureas, nucleobases, steroids, dendrimers, etc. [13]). Although a great effort has been made to investigate the structure–property relationships, it is still impossible to design a new LMOG de novo. For those reasons most of the known LMOGs have been discovered serendipitously. Nevertheless, with the knowledge gained about the mode of aggregation of LMOG molecules some of the structural features necessary for gelation are known. The presence of a supramolecular synthon to promote the one-dimensional (1D) self-assembly is a necessary feature in order to form the fibrillar entangled network that entraps the solvent [14]. The strongest and most important supramolecular synthons involve functional groups that possess a complementary donor–acceptor hydrogen bond motif, such as for instance amides, ureas, carbamates, saccharides, ammonium carboxylate salts, etc. A rod-like molecular shape is also a general structural requirement for steroid derived LMOGs because it allows a good face to face molecular contact to promote the one-dimensional growth. These concepts have been recently exploited to design new LMOGs [15–16]. Nevertheless, the presence of a supramolecular synthon in a molecule is a necessary but not a sufficient feature to become an organogelator. The formation of the gel involves a delicate balance of cooperative forces between the directional self-assembly that promotes the 1D aggregation and the solubility and insolubility in a given solvent, which is based on the specific interactions between solvent and gelator molecules [17]. Numerous attempts have been made to correlate solvent parameters to gelation ability. The most promising technique was recently presented in the works of Bouteiller et al. and Rogers et al., in which they apply the Hansen solubility parameters (HSP) to evaluate the gelation behavior of LMOGs in different solvents [18–19]. The Kamlet–Taft solvatochromic parameters, which consider separately the hydrogen-bond donor (HBD, α), hydrogen-bond acceptor (HBA, β), and polarizability (π*) properties as contributions to the overall solvent polarity had also been occasionally used to study solvent–gelator specific interactions [20–21].

LMOGs based on cholesterol and bile acids offered the most versatile units on which to base the systematic design of functional LMOGs for the gelation of organic solvents. Neither cholesterol nor cholestanol are gelator molecules, and although a variety of steroid derivatives has been analyzed over the years only a few simple analogues are known to be organogelators [22]. The steroidal LMOGs usually have substituents attached to the 3β-OH of the cholestane A ring and synthetic variations at the steroidal skeleton are scarce. Cholesterol-based LMOGs build mesophases in which steroid–steroid stacking is controlled by van der Waals forces. These interactions, including the additional intermolecular contacts from the pending moieties linked at C-3 (usually hydrogen bond and π–π stacking), lead to a primarily one-dimensional long-range growth and finally produce the interconnected 3D self-assembled fibrillar network (SAFIN), which traps the solvent and turns into a self-supporting gel. There are several types of cholesterol-based LMOGs grafted on hydrophilic heads such as saccharides, chromophores, ligands, peptides, etc. The first study on rational syntheses of cholesterol derived organogels was made by Weiss et al. [23] on a family of molecules containing an aromatic moiety (A) connected to a steroidal group (S) through a linker (L). Since then a great number of ALS molecules have been discovered constituting the most systematically investigated family of LMOGs [24].

**Figure 1:**
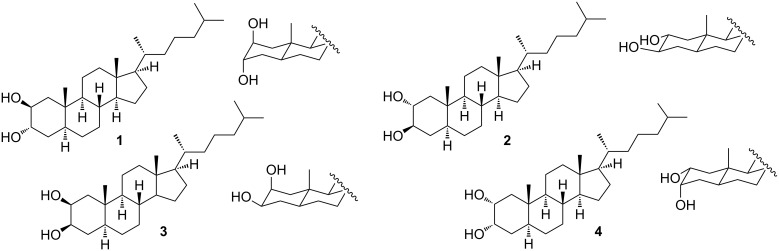
Structures of the 2,3-dihydroxycholestane isomers studied in this work.

Recently we have reported a new steroid-based organogelator with a non-conventional bridged pregnane skeleton and its use as template for the preparation of nanotubes and fluorescent nanospheres of silica via the in-situ sol-gel polymerization of tetraethyl orthosilicate (TEOS) [25–26]. In our search for new LMOGs analogues with structural variations in the steroidal nucleus and new properties we prepared steroid **1** as a synthetic intermediate (Figure 1). This known steroid, which is widely used in medicinal chemistry as a precursor in the synthesis of cholesteryl derived bioactive analogues [27–28], gelated *n*-hexane during concentrating the fractions from a chromatographic column. Although this simple steroid has been synthesized for the first time decades ago, its gelation property has never been described in the literature.

In this paper, we report the broad scope and super-organogelating ability of steroid **1** and the use of their gels as template for the in situ polymerization of TEOS. A general self-assembly model with multiple intermolecular hydrogen bond interactions is proposed based on experimental and computational data. In order to understand the role of the *trans*-diaxial orientation of the vicinal dihydroxy moiety we have also studied the organogelating properties of the stereoisomers **2**–**4** bearing equatorial hydroxy groups (Figure 1). The gelation ability of **1** is discussed in terms of the Hansen solubility parameters and Kamlet–Taft parameters based on its behavior in a set of 33 solvents. We also report on the sol–gel polymerization of TEOS carried out with gels of **1** demonstrating that templated silica nanotubes are obtained only under controlled conditions. Considering that the design of new LMOGs with predictable gelation properties is still a challenge nowadays, we consider that the *trans-*diaxial dihydroxy supramolecular synthon studied herein is a valuable contribution towards the development and design of new LMOGs molecules with potential applications.

## Results and Discussion

As mentioned before, steroid **1** showed an excellent gelation ability in *n*-hexane during concentrating a solution on a rotatory evaporator. Preliminary qualitative tests with cyclohexane and dichloromethane also showed a good gelling ability. These remarkable properties for such a simple steroid molecule prompted us to study the gelation scope, morphology, mode of self-assembly and the potential use of the gels to prepare silica nanoparticles through a bottom-up approach.

### Gelation scope and thermal stability

To assess the scope of the gelation ability of steroids **1**–**4** in a simple way, the test tube method was first used with 28 selected organic solvents ranging from hydrocarbons to alcohols (Table 1, entries 1–28). While steroids **2**–**4** were unable to gelate any of these solvents, steroid **1** showed a good to excellent gelation ability over a wide variety of solvents.

**Table 1:** Gelation test for LMOG **1**.

entry	solvent	test^a^	CCG^b^ (wt %)

1	1,2-dicloroethane	G	1.4
2	1,4-dioxane	G	7.0
3	1-hexanol	S	—
4	acetic acid	S	—
5	acetone	I	—
6	acetonitrile	I	—
7	aniline	G	2,0
8	CCl_4_	G	0.8
9	chloroform	G	2.5
10	pyridine	S	—
11	dichloromethane	G	0.8
12	DMF	S	—
13	DMSO	G	5.0
14	ethanol	I	—
15	ethyl acetate	S	—
16	isopropyl ether	I	—
17	methanol	I	—
18	*n*-butanol	I	—
19	*n*-heptane	TG	0.06
20	*n*-hexane	TG	0.06
21	cyclohexane	TG	0.13
22	TEA	I	—
23	TEOS	S	—
24	THF	S	—
25	toluene	G	2.5
26	water	I	—
27	xylene	G	0.8
28	methyl acrylate	G	4.0
29	nitrobenzene	G	3.3
30	methylcyclohexane	TG	0.16
31	decane	TG	0.04
32	styrene	G	2.5
33	*o*-dichlorobenzene	G	5.0

^a^G: gel, TG: turbid gel, S: soluble I: insoluble; ^b^critical concentration for gelation.

**Figure 2:**
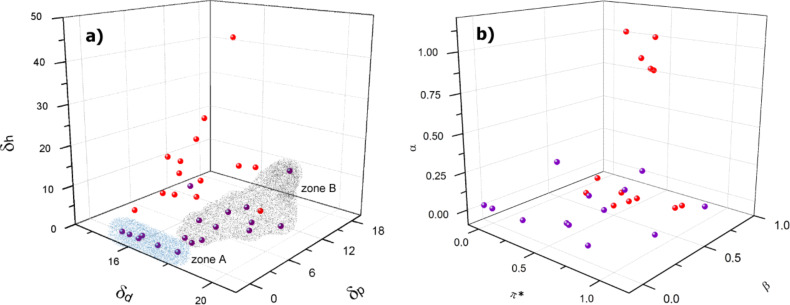
3D plots for LMOG **1** and solvent parameters of the tested solvents a) Hansen solubility parameters (δ_d_ dispersive interactions, δ_p_ dipolar interactions, δ_H_ hydrogen bonding) and b) Kamlet–Taft parameters (α hydrogen bond donor, β hydrogen bond acceptor, π^*^ polarizability). Purple: gelated solvents. Red: non-gelated solvents. (For more details see Table S1 in Supporting Information File 1.)

Given these results we directed our analysis towards the scope of solvent gelation of **1** by using the HSP approach described by Boutellier [18]. A tridimensional plot of the dispersive interactions (δ_d_), the dipolar-interactions (δ_p_) and the hydrogen-bonding (δ_H_) parameters of LMOG **1** showed a complex correlation between the gelation ability and the HSP of the solvents (Figure 2, see Supporting Information File 1 for full data). Two gelation zones were clearly identified graphically. The first gelation space (zone A), with a cylindrical, almost linear profile, involves solvents with zero or very low δ_H_ and δ_p_ parameters and a δ_d_ parameter between 14 and 18 (hydrocarbons). Gelation zone B includes polar, non-protic solvents with higher dispersive interaction parameters δ_d_ (from 17.5 to 20). In contrast to the results obtained by Boutellier et al. [18], it was not possible to define a gelation sphere in either zone in this case. Nevertheless, in order to corroborate the tendency observed we chose a new set of solvents with HSP between the gelation spaces (Table 1, entries 29–33), all of them could be gelated by **1** showing that, although spherical spaces cannot be defined, prediction is possible by selecting solvents with HSP inside the gelation zones*.* Figure 2 qualitative shows that there is a good correlation between the HSP and the gelated solvents, pyridine and methyl acrylate are the only solvents that visible lay outside the corresponding zones. It is clear from the graphic analysis that the higher the δ_H_, the higher the δ_p_, of the gelated solvent with a limit of about 10 for the former. Non-gelled solvents are clustered in zones with high δ_H_ and low δ_d_. This makes sense, because a solvent, which acts as either a strong hydrogen-bond donor or a strong hydrogen-bond acceptor, will significantly interact with the hydroxy groups of **1** and thus impede the supramolecular self-assembly and the formation of the gel. This way it will prevent gelation or will make a higher concentration of gelator necessary. This effect is higher for solvents with smaller dispersive interaction parameters. For solvents in zone A steroid **1** is a supergelator with critical concentrations for gelation (CCG) below 0.1 wt % for linear hydrocarbons (entries 19, 20 and 31) and of about 1% for cyclic hydrocarbons and carbon tetrachloride (entries 8, 21 and 30). The second zone comprises aromatic and polar solvents bearing nitrogen or oxygen atoms with CCG values between 2 and 7 wt %. The presence of two gelation zones is indicative of a difference in the molecular packing of the gel fibers and will be discussed in the following section.

Next we considered the Kamlet–Taft parameters for the tested solvents and compared these results to the Hansen approach [29]. The 3D plot did not show clear zones of gelation (Figure 2b) although it is evident from the analysis that solvents with very high α parameters (α > 0.5, such as alcohols, water and formic acid) cannot be gelated. The same tendency is observed for the β parameter but with some exceptions, such as DMSO, which is a very strong hydrogen-bond acceptor but still capable of forming gels. The effect of π* seems to be less important since solvents with both high and low polarizability can be gelled (see π*-vs-α and π*-vs-β plots in Supporting Information File 1). We assume that the bad correlation between the Kamlet–Taft parameters and the gelation behavior of LMOG **1** is connected to the solvatochromic origin of the scale [29]. The Hansen solubility parameters with a scale based on solubility seems to be more suitable for solvent–gelation analysis.

**Figure 3:**
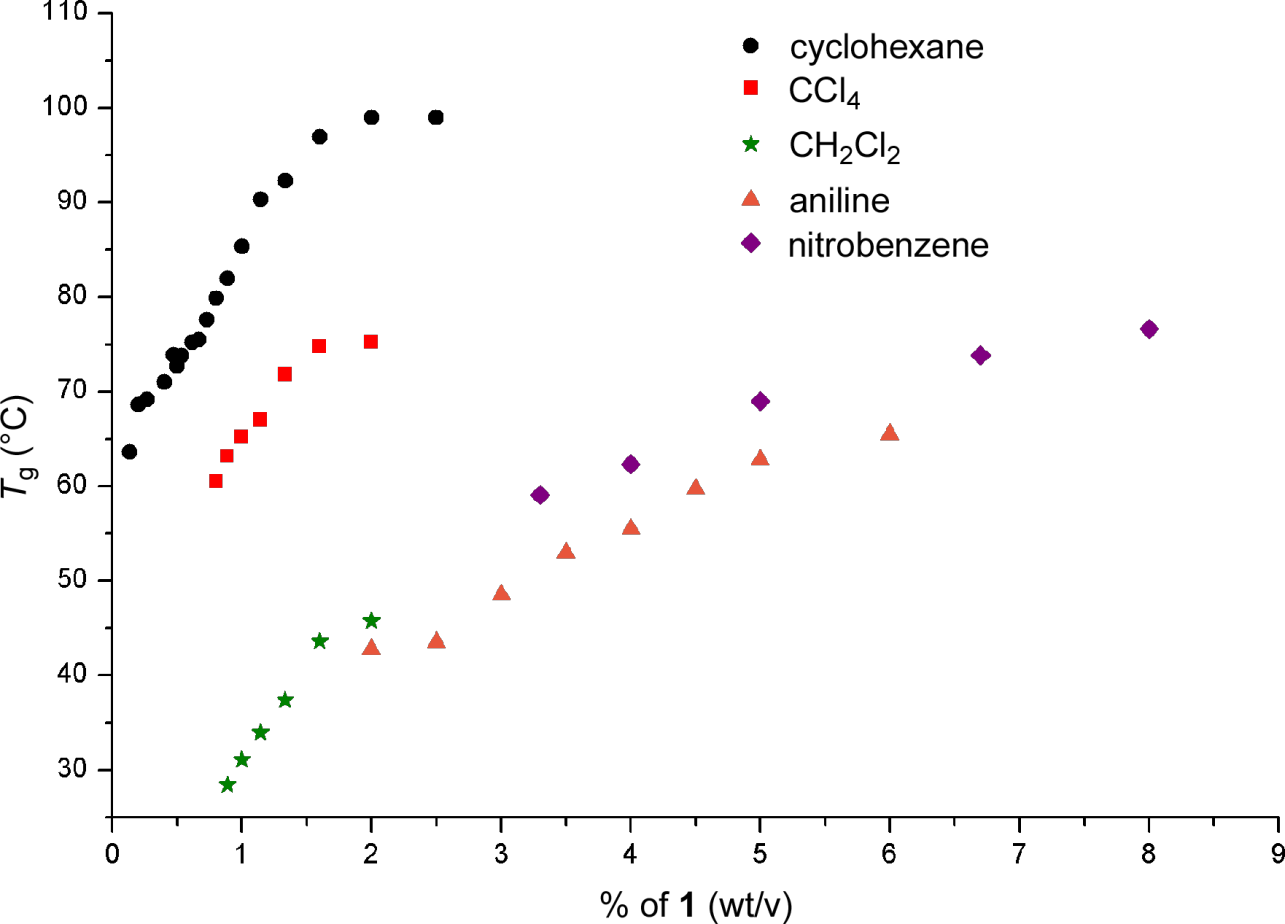
*T*g-vs-concentration plots for gels of **1**.

All gels were stable, thermoreversible and non-thixotropic. Gels from hydrocarbons were turbid, depending on the concentration of gelator, indicating a low solubility. To estimate the relationship between thermal stability, concentration of gelator, and solvent we studied the variation of the gelation temperature (*T*g) with the concentration of **1** in gels of cyclohexane, dichloromethane, carbon tetrachloride, dioxane, aniline and nitrobenzene. Tube inversion experiments were performed to measure *T*_g_. This method was selected because of its simplicity and widespread use in the field of gel-phase materials. Typically, as the concentration of **1** was increased, *T*_g_ also increased until a plateau region was reached. Cyclohexane gave the most stable gel with a *T*_g_ of 64 °C at the CCG, and a maximum *T*_g_ value of 99 °C for a 2-wt-% gel (Figure 3). Carbon tetrachloride and dichloromethane reached the plateau region at the same concentration, but with *T*_g_ values of 75 and 45 °C, respectively, which shows that more polar solvents gives thermally less stable gels. On the other hand, dioxane, nitrobenzene and aniline, capable of hydrogen bonding, had a maximum *T*_g_ of 72 °C, 77 °C and 66 °C, respectively, but at considerably higher concentrations of 26, 6 and 8 wt % (see Supporting Information File 1). LMOG **1** could also selectively gelate the organic layer from a water/organic solvent mixture after a heating–cooling process.

### Morphology and self-assembly

The FTIR spectra of the gel and the solution of LMOG **1** provided evidence that the interactions leading to self-assembly is primarily hydrogen bonding between the hydroxy groups. In dichloromethane (DCM) solution, at a concentration of LMOG **1** below the CCG, a broad band was observed at ν_O–H_ = 3608.2 cm^−1^ (O–H stretching). In the gel state this band was widened and shifted to 3363.3 cm^−1^, which is typical for an intermolecular hydroxy hydrogen-bond. The FTIR spectrum of the gel still showed the band of free hydroxy groups at ν_O–H_ = 3610.1 cm^-1^ and may be associated with molecules of LMOG **1** in the liquid-like solution phase trapped within the SAFIN (see Supporting Information File 1 for IR spectra) [17].

**Figure 4:**
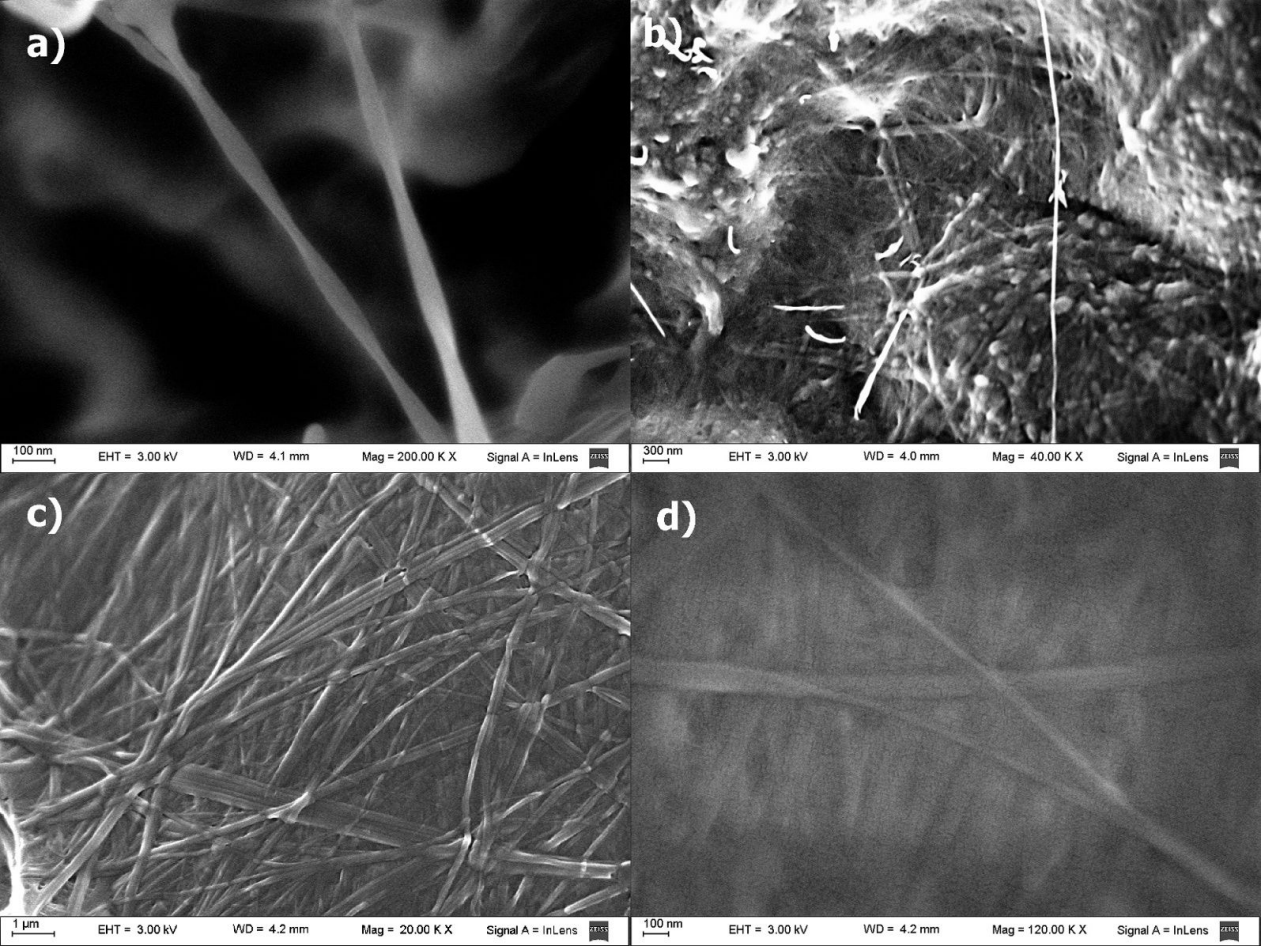
SEM images of xerogels from a,b) dichloromethane, and c,d) from dioxane.

The microscopic morphology of the xerogel of **1** from DCM, *n*-hexane and dioxane was analyzed by SEM. The images showed an entangled fibrillar network for all solvents. Particularly the images of the dichloromethane xerogel (Figure 4a and Figure 4b) showed left handed helical fibers with a fiber width ranging from 20 to 75 nm, and a helicoidal period of about 750 nm. The helical shape is clearly observed in the isolated fibers (Figure 4a), but these are difficult to find in the bulk due to the collapsed fibrillar network. For *n*-hexane and dioxane simple straight entangled fibers were observed with a minimum width of 20 nm. In all cases fibers with lengths of up to several micrometers were clearly visible. SEM images of the xerogel from dioxane showed a tighter SAFIN compared to *n*-hexane and dichloromethane xerogels. This is indicative of a more compact assembly in this polar solvent. Due to the lack of a chromophore in **1**, it was not possible to use circular dichroism to prove the helicoidal nature of the fibrillar network, but images suggest that, at least in dichloromethane, the one dimensional self-assembly is directed helicoidally by the inherently asymmetric steroid molecule.

**Figure 5:**
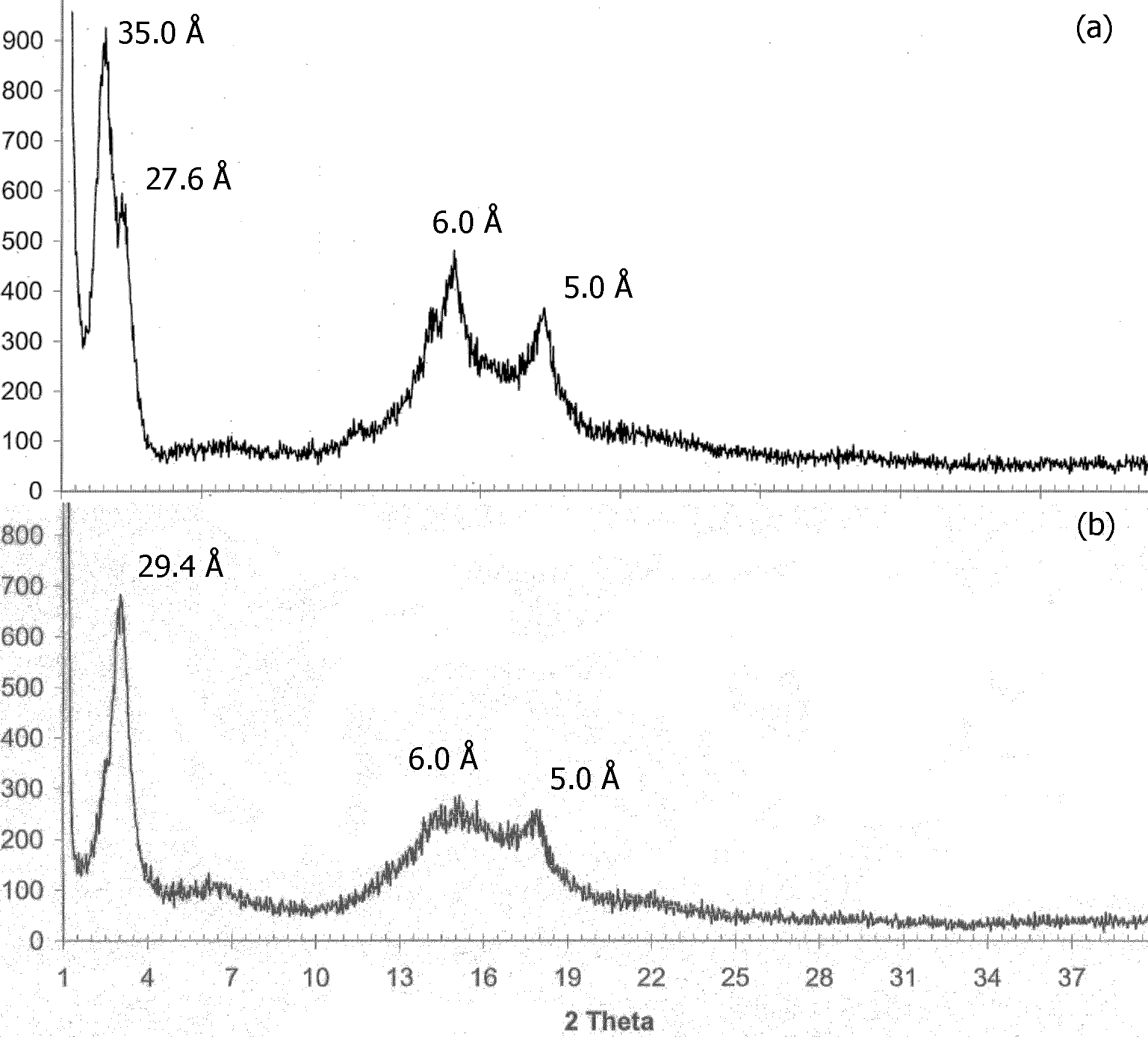
Powder X-ray diffraction pattern of the xerogels of **1** from a) *n*-hexane and b) dichloromethane.

**Figure 6:**
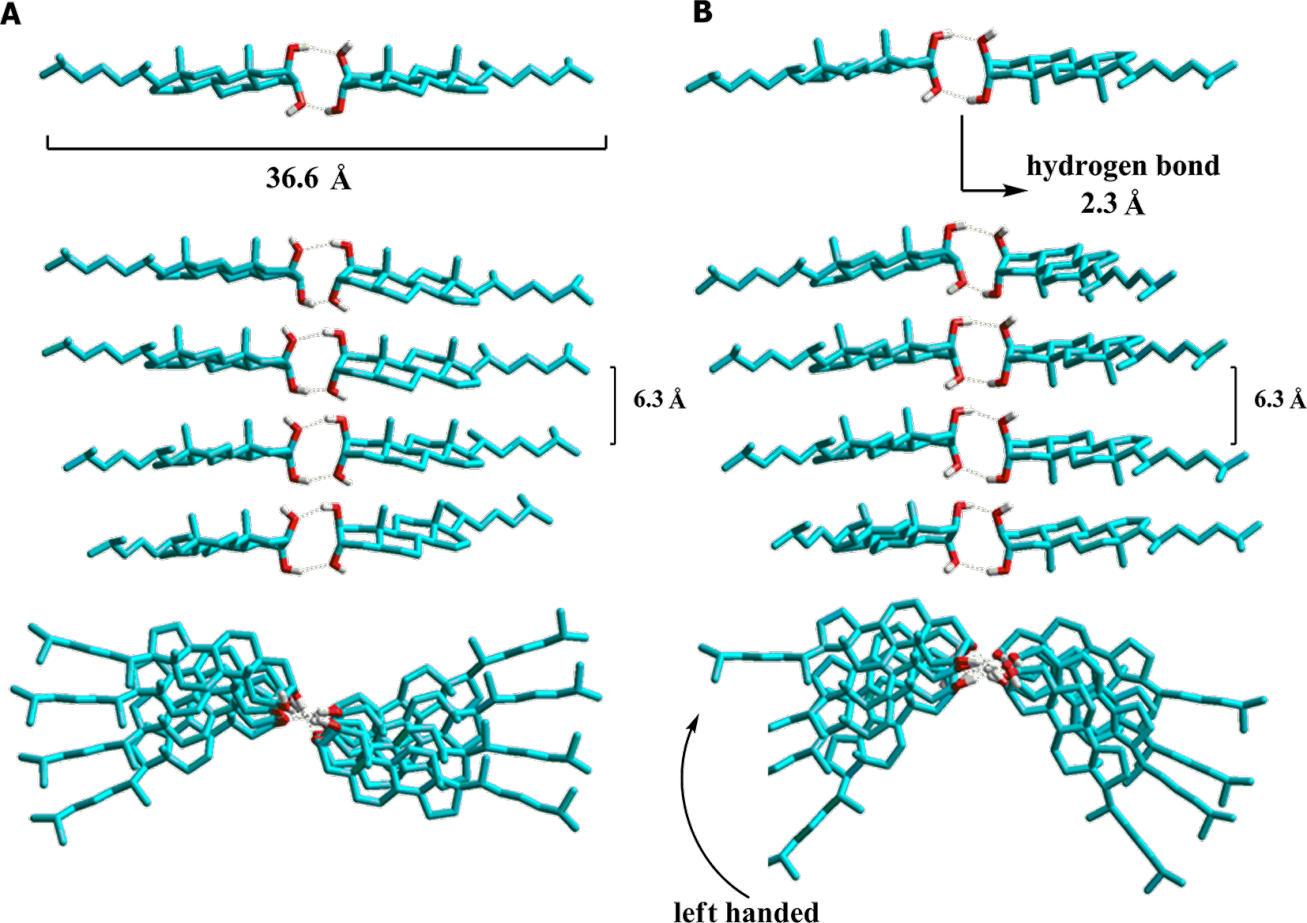
Self-assembly models proposed for LMOG **1**, only the left handed helix is shown, head to head hydrogen bonds are shown in dotted lines, hydrogen atoms have been removed for clarity. System **A**: hydrogen bonds at C2–OH/C2′-OH and C3–OH/C3′–OH; system **B**: C2–OH/C3′–OH and C2′–OH/C3–OH.

To gain a better insight into the packing of the material, X-ray powder diffractograms (XRPD) of xerogels from dichloromethane (DCM) and *n*-hexane were performed (Figure 5). The X-ray pattern of the xerogel of **1** from *n*-hexane showed an intense scattering peak at *d* = 35.0 Å with a small shoulder at 27.6 Å and two smaller and broader peaks at *d*= 6.0 and 5.0 Å (Figure 5a). The larger *d* value can be associated to the repeating distance of the aggregates. In the case of the xerogel from DCM this peak appears at *d* = 29.4 Å, and the generally less sharp peaks indicate a less ordered self-assembly (Figure 5b). As shown in Figure 6, the scattering peak at *d* = 35 Å of the *n*-hexane gel perfectly correlates with the distance of two molecules of **1** arranged head-to-head with completely elongated side chains. Taking this into account the specific molecular packing of gels of **1** may be understood from its molecular structure itself. Steroid **1** is a typical amphiphilic compound in which the dihydroxy moiety defines a hydrophilic head while the rest of the skeleton (tail) is hydrophobic. The amphiphilic property causes molecules to aggregate into structures that avoid the unfavorable head-to-tail contact, and so, head-to-head and tail-to-tail contacts are directing the self-assembly. In more polar solvents such as DCM, the side chain of the steroid is not fully elongated to avoid the interaction of DCM molecules with the polar head. This is evidenced by the shift of *d* from 35.0 Å (*n*-hexane) to 29.4 Å (DCM). On the other hand, the spatial orientation of the hydroxy groups is critical since only steroid **1**, with both hydroxy groups at axial positions, has the ability of a long range self-assembly to promote gelation. To get an insight of the necessary molecular spatial requirements for the 2,3-dihydroxy moiety to reach an optimal hydrogen bonding, we studied the possible mode of self-assembly of the four isomers by a molecular modeling simulation in vacuum (semiempirical AM1).

A conformational study of a single molecule of LMOG **1** (semiempirical, AM1) showed a distance of 18.0 Å for the molecule with the elongated side chain (Figure 6) suggesting a repetitive unit involving a head-to-head self-assembly between two molecules, as explained above. Next, we minimized different modes of head-to-head interactions and found two possible arrangements with minimal energies. In the first one, both molecules interact with the α faces of the steroids pointing to the same side (Figure 6, system **A**). In the second case the α faces are oriented to opposite sides (Figure 6, system **B**). Both dimers were similar in energy with stabilizations of 9.7 and 10.0 kcal/mol compared to the isolated molecules. This stabilization energy arises from the two intermolecular hydrogen bonds between the hydroxy groups with a H–OH distance of 2.4 Å. We then analyzed the one-dimensional arrangements of the dimers described above by an α- and β-helix self-assembly. We placed the dimers in a way so that the central hydrophilic zones can interact with each other with a distance of 2.7 Å between the hydroxy groups (to allow hydrogen bond interactions) and a rotation angle around the hydrogen bond axis of ±18 degrees (corresponding to left- and right-handed helices, respectively). After minimization, the left handed assemblies were slightly more stable than the right handed, but with no significant differences (0.2–0.3 kcal/mol). For this reason we will only discuss the energy of the left-handed systems. The stabilization energy for each dimer–dimer interface for the β-octamer **A** was 8.1 kcal/mol and for the β-octamer **B** 8.3 kcal/mol. This stabilization between the dimers arises from hydrogen bonding between the dimers with H–OH distances of about 2.8 Å, and van der Waals interactions between the hydrocarbonated skeletons of the steroid.

A structural analysis of the steroids **2**, **3** and **4**, with hydroxy groups at equatorial positions, showed that angles and distances between the hydroxy groups are inadequate to give the intermolecular hydrogen bonding necessary for the one-dimensional self-assembly. These results led to the conclusion that for LMOG **1**, the arrangements proposed are valid modes of self-assembly in agreement with the experimental results. Both, left- and right-handed self-assembled systems, are stable at the conditions of the calculations and this stabilization arises from the multiple hydrogen bonds that are only allowed for the *trans*-diaxial dihydroxy system present in organogelator **1**. As mentioned before, the existence of two clearly differentiated gelation zones in the HSP plot is indicative for a difference in the molecular packing of the gel fibers in non-polar and non-protic polar solvents. In the last case the packing is tight enough to prevent the polar solvent molecules to interact with the polar head of the steroid breaking the hydrogen bonds that lead to the SAFIN.

### Templated preparation of silica nanoparticles

**Figure 7:**
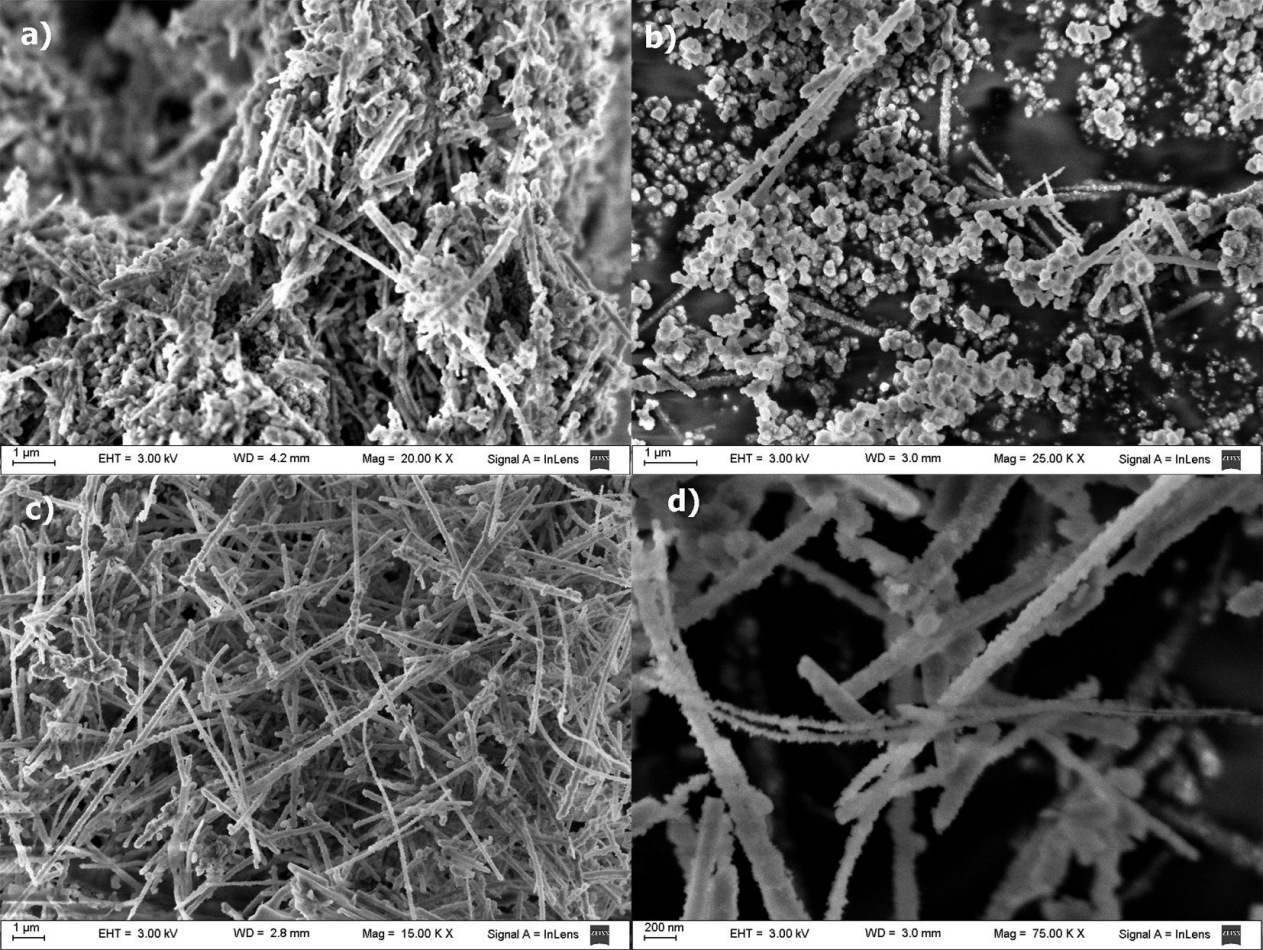
SEM images of nanostructured silica obtained from gels of LMOG **1** under the following conditions: 0.5 mL of dioxane, 0.1 mL TEOS, 35 mg of **1**, and (a) 3 μL of benzylamine and 15 μL of water, (b) 3 μL of benzylamine and 3 μL of water (c,d) 1 μL of benzylamine and 15 μL of water.

Even though LMOG **1** does not gelate tetraethoxysilane (TEOS), in-situ sol–gel polymerization experiments were performed with dioxane and dichloromethane gels containing 16.6% of TEOS with benzylamine as a catalyst. The morphology of the nanostructured silica obtained was analyzed by SEM microscopy (Figure 7). A first polymerization attempt using 3 μL of catalyst and 15 μL of water showed a mixture of amorphous silica with nanotubes in a 3/2 ratio reflecting a partial template polymerization process (Figure 7a). Since high reaction rates usually disfavor the template process for the sol–gel polymerization of TEOS, we decided to slow down the reaction rate by lowering the water concentration and the catalyst load. In the first case (Figure 7b) a highly amorphous material was observed, while lowering the benzylamine load to a third rendered only nanotubes of silica with external diameters between 50 and 175 nm and lengths of several micrometers (Figure 7c,d). Lowering the catalyst load down to a tenth rendered a higher amount of amorphous material. To assess the role of the fibrillar network in the templating process we repeated the experiment under the same conditions, in which nanotubes were obtained, but in absence of gelator. In this case only amorphous silica was observed by SEM microscopy of the product (see Supporting Information File 1), which proves the directing role of the fibers during the polymerization of TEOS. Next we performed the polymerization of TEOS in dichloromethane gels to confirm the helicoidally nature of the fibers by templation. The SEM images of the product obtained showed spherical nanoparticles of irregular size (see Supporting Information File 1). In this solvent the templated sol–gel polymerization failed, but the formation of spherical particles indicates some control effect on the growth of the silica nucleus by the fibrillar network of the gel. Tubular nanostructured materials, such as these presented here, may offer alternatives over spherical nanoparticles for some biomedical and biotechnological applications [30–31].

## Conclusion

In summary, we have studied the organogelating behavior of the four stereoisomers of the structurally simple 2,3-dihydroxycholestane. The theoretical and experimental results on the four stereoisomers indicate that the *trans*-diaxial orientation of the hydroxy groups on this supramolecular synthon is essential for the gelating property. Only the isomer with the *trans*-diaxial dihydroxy group had the ability to gelate a wide variety of organic solvents and to be a superorganogelator for hydrocarbons with a minimal concentration for gelation of 0.04 wt %. From the analysis of the solvent parameters we conclude that the Hansen solubility parameter (HSP) approach is more suitable to study the solvent–gelation relationship than the solvatochromic Kamlet–Taft scale. The HSP analysis showed two gelating zones indicative of different packing in polar and non-polar solvents. In contrast to the results obtained by Boutellier et al., it was not possible to define a gelation sphere in either zone, but a qualitative analysis showed that predictions are still possible for this complex system. The FTIR, XRPD, and semiempirical molecular modeling studies allowed us to propose a packing mode in which the amphiphilic steroid self-assembles in a head-to-head mode through two hydrogen bonds between the dihydroxylic system. The resulting dimers can then form 1D aggregates, in which multiple hydrogen bonding plays an important role together with van der Waals interaction stabilization. In case of polar solvents, capable of hydrogen bond formation, the packing of LMOG **1** is tighter in order to prevent the solvent molecules to interact with the polar head of the steroids, which would break the self-assembled fibrillar network. Finally, the dioxane gel was successfully used as template to grow silica nanotubes through sol–gel polymerization of TEOS under basic catalysis. We conclude that the success of the template on dioxane strongly depends on the catalyst load. Usually the structural motif or element enabling the more efficient transcription for template synthesis of inorganic oxides involves a covalently attached positive charge. For neutral organogelator **1**, electrostatic or hydrogen-bond interactions between the intermediate anionic silicate species and the fibrillar network may be proposed as the only driving force directing the template. Such nanostructured materials may have biomedical and biotechnological applications that offer alternatives over spherical nanoparticles. Among the solvents gelled by steroid **1**, styrene and methyl acrylate offer great potential in the preparation of mesoporous polymers. We are currently exploring these materials and their potential applications.

## Experimental

### Materials

Cholesterol (94%) was purchased from Sigma-Aldrich. *n*-Hexane, ethyl acetate, dichloromethane and THF were fractionally distilled, and the remaining solvents were used as supplied by the manufactures.

### Methods

**Synthesis***:* 2β,3α-dihydroxycholestane (**1**), 2α,3β-dihydroxycholestane(**2**), 2β,3β-dihydroxycholestane(**3**) and 2α,3α-dihydroxycholestane (**4**) were prepared from cholesterol following the procedures described in the literature [27]. The identities of compounds **1**, **3** and **4** were confirmed by comparing the NMR spectra with those found in the literature. ^13^C NMR spectra of compound **2** did not match the literature data [27–28] and was completely characterized to confirm its identity concluding that the ^13^C NMR data reported in literature was mistaken (see Supporting Information File 1).

**Gelation Tests:** The test were carried out in a similar manner as described in [25]. The gelation ability was investigated by a typical test tube experiment. A mixture of a defined amount of gelator and a volume of the solvent (10% wt/v) in a closed flask was heated and shaken until the solid was dissolved and then slowly cooled to room temperature. If a stable gel was observed after inversion of the flask, it was considered a gel (G). When gelation was not observed at room temperature, the sample was cooled at 5 °C. The critical concentration for gelation (CCG) was determined by subsequent dilution of the original organogel followed by a heating–cooling process until gel formation was not observed at room temperature (20 °C)

The reversible gel–sol transition temperatures (*T*_g_) were measured using the classical inverted tube method [32].

**Phase-selective gelation experiment:** Compound **1** (8 mg) was added to a flask with a mixture of 1 mL of dichloromethane and 1 mL of water, the flask was closed, shaken and heated until the solid was dissolved. Then, the solution was cooled and left at room temperature. After ca.15 min the dichloromethane phase became a gel and the water layer was still fluid.

**Xerogel preparation**: The preparation was carried out in a similar manner as described in [25]. The xerogels were prepared by cooling the gels in a bath at −90 °C, evaporating the solvent under high vacuum over 6 h and then slowly letting the gels get to room temperature under vacuum.

**FTIR measurements:** The measurements were carried in a similar manner as described in [25]. Fourier transform infrared (FTIR) measurements of the solution and the gels of **1** were performed on a Nicolet Magna IR 550 FTIR spectrometer in a demountable liquid cell with two NaBr disks, 32 mm in diameter and a 0.5 mm thick Teflon spacer. For the dichloromethane gel, a warm solution of **1** (0.25 wt %) was injected into the cell and allowed to cool down for 10 min at room temperature before measuring the spectra.

**X-ray powder diffraction measurements:** Diffraction patterns were obtained by using a Siemens D5000 diffractometer with Cu Kα radiation (λ = 1.54056 Å), the stepsize was 0.025° with a measurement time of 6 s per step.

**Scanning electron microscopy:** SEM measurements were performed in a similar manner as described in [25]. SEM pictures of the xerogel and silica nanoparticles were taken on a Carl Zeiss NTS SUPRA 40FEG scanning electron microscope. A small portion of the solid sample (xerogel or silica) was attached to the holder by using a conductive adhesive carbon tape. Prior to examination the xerogels were coated with a thin layer of gold.

**Sol–Gel polymerization of TEOS in dioxane:** Compound **1** (35 mg) was dissolved by heating and shaking in dioxane (0.5 mL) and TEOS (0.1 mL) with an addition of benzylamine (0.1–3.0 μL) and water (3–15 μL). The solution was cooled down to room temperature until gelation was observed and then left at room temperature for 6 days. Subsequently, the sample was diluted in dichloromethane, the solid was centrifuged, and washed once with dichloromethane. The silica was heated at 200 °C for 2 h and 600 °C for 4 h in air.

**Molecular modeling experiment**: These experiments were conducted in a similar manner as described in [25]. The computational experiments were performed with HyperChem 8.0.4, semiempirical optimization, AM1 method in vacuum. Algorithm: Fletcher–Reeves. Termination condition, RMS gradient: 0.05 kcal/(Å·mol). No bond or distance restrictions were imposed. The interaction energies were estimated from the difference between the heats of formation of the different arrangements divided by the number of molecule–molecule interfaces. To have an insight in the stabilization energy of the 1D self-assembled models proposed we carried out the following experiments: Experiment I: an isolated molecule of LMOG **1** was minimized using the above conditions; Δ*H*_f_ = −201.46 kcal/mol. Experiment II: head-to-head dimers of LMOG **1** were minimized, the molecules were placed facing the hydroxy groups at a distance typical for hydrogen bonds: Dimeric system **A**: C2-OH/C2′–OH and C3–OH/C3′–OH; ΔH_f_ = −412.60 kcal/mol. Dimeric system **B**: C2–OH/C3′–OH and C2′–OH/C3–OH; Δ*H*_f_ = −412.91 kcal/mol. Experiment III: four dimers from experiments II were placed in a 1D arrangement facing the α- and β-faces of the steroids with a separation of about 6 Å between the steroid skeleton, and a rotation angle around the hydrogen bond axis of +18° and −18° (α- and β-helix). No bond or distance restrictions were imposed. The right and left handed helix of systems **A** and **B** gave similar heats of formation with no significant differences. Octameric system **A** (left handed helix): Δ*H*_f_ = −1674.52 kcal/mol; octameric system **B** (left handed helix): Δ*H*_f_ = −1676.62 kcal/mol. The stabilization energy for the head-to-head interaction in each molecule–molecule interface was estimated from the difference between the heats of formation of the dimers and the isolated molecules. The stabilization energy for the dimer–dimer interaction in the one dimensional arrangement interface was estimated from the difference between the heats of formation of the octamers and the dimers.

## Supporting Information

Supporting Information features additional experimental data, i.e. characterization data of steroid **2**, SEM images, *T*_g_-vs-concentration plots, FTIR spectra, and HSP plots.

File 1 Additional experimental data.
