# Supplementary material for: Self-assembly of 2,3-dihydroxycholestane steroids into supramolecular organogels as a soft template for the in-situ generation of silicate nanomaterials
Source: Beilstein J Org Chem. 2013 Sep 9;9:1826–36. doi: 10.3762/bjoc.9.213 (PMC3778393; doi:10.3762/bjoc.9.213)
Supplement: File 1 — Additional experimental data. [file Beilstein_J_Org_Chem-09-1826-s001.pdf]

Supporting Information

for

Self-assembly of 2,3-dihydroxycholestane steroids into supramolecular organogels as a soft template for the in-situ generation of silicate nanomaterials

Valeria C. Edelsztein, Andrea S. Mac Cormack, Matías Ciarlantini and Pablo H. Di Chenna*

Address: Departamento de Química Orgánica and UMYMFOR (CONICET-FCEN), Facultad de Ciencias Exactas y Naturales, Universidad de Buenos Aires, Ciudad Universitaria, Pabellón II, Buenos Aires, C1428EGA, Argentina

Email: Pablo Héctor Di Chenna* - dichenna@qo.fcen.uba.ar
* Corresponding author

Additional experimental data

Table of contents:

Characterization of 2α,3β-dihydroxycholestane (**2**)...……………………………………S2

NMR data for steroids **1**, **3** and **4**……..…………………………………………………S6

HSP parameters..…………………...……………………………………………………..S9

HSP plots…………………………..……………………………………………………….S10

Kamlet–Taft parameters.………….…………………………………………………..S11

T_g-vs-concentration plots……….……….……………………………………………S12

FTIR spectra……………..……………………………………… ……… ……………..S13

SEM images of xerogels of **1**…..……………………………………………………..S14

SEM images of silica nanoparticles………………………… ……….…………S14

Characterization of 2α,3β-dihydroxycholestane (2)

The structure and stereochemistry of this compound were completely characterized by 1D and 2D NMR spectroscopy (HSQC, HMBC, COSY and NOESY)

^{1}H NMR ($CDCl_3$, 500 MHz) δ 3.58 (m, 1H, H-2β), 3.39 (m, 1H, H-3α), 1.98 (m, 3H, H_2-12 and H-1β), 1.80 (m, 1H, H-6), 1.64 (m, 2H, H-4α and H-7), 1.51 (m, 3H, H_2-25 and H-23), 1.22 (m, H-5α), 0.95 (m, H-1α), 0.90 (d, *J* = 6.5 Hz, 3H, H-21), 0.86 (d, *J* = 6.7 Hz, 3H, H-26), 0.85 (d, *J* = 6.7 Hz, 3H, H-27), 0.84 (s, 3H, H-19), 0.70 (dt, *J* = 4.0; 12.4 Hz, 1H, H-14), 0.64 (s, 3H, H-18) ppm.

^{13}C NMR ($CDCl_3$, 125 MHz) δ 76.48 (C-3), 73.12 (C-2), 56.32 (C-17), 56.24 (C-14), 54.30 (C-), 45.08 (C-1), 44.86 (C-5), 42.59 (C-13), 39.92 (C-12), 39.50 (C-24), 37.48 (C-10), 36.15 (C-22), 35.78 (C-20), 35.61 (C-4), 34.77 (C-8), 31.90 (C-7), 28.23 (C-6), 28.00 (C-25), 27.94 (C-16), 24.19 (C-23), 23.82 (C-15), 22.81 (C-26), 22.55 (C-27), 21.38 (C-11), 18.66 (C-21), 13.51 (C-19), 12.06 (C-18) ppm.

HRMS (ESI): calcd. ($C_{27}H_{48}O_2$+Na^+) 427.3543; found: 427.3542; mp (from hexane/ethyl acetate): 204–206 °C (lit. 204 °C); FTIR: ν_{max} (cm^{-1}) 3355.1; 2927.8; 2841.3; 1454.6; 1040.4

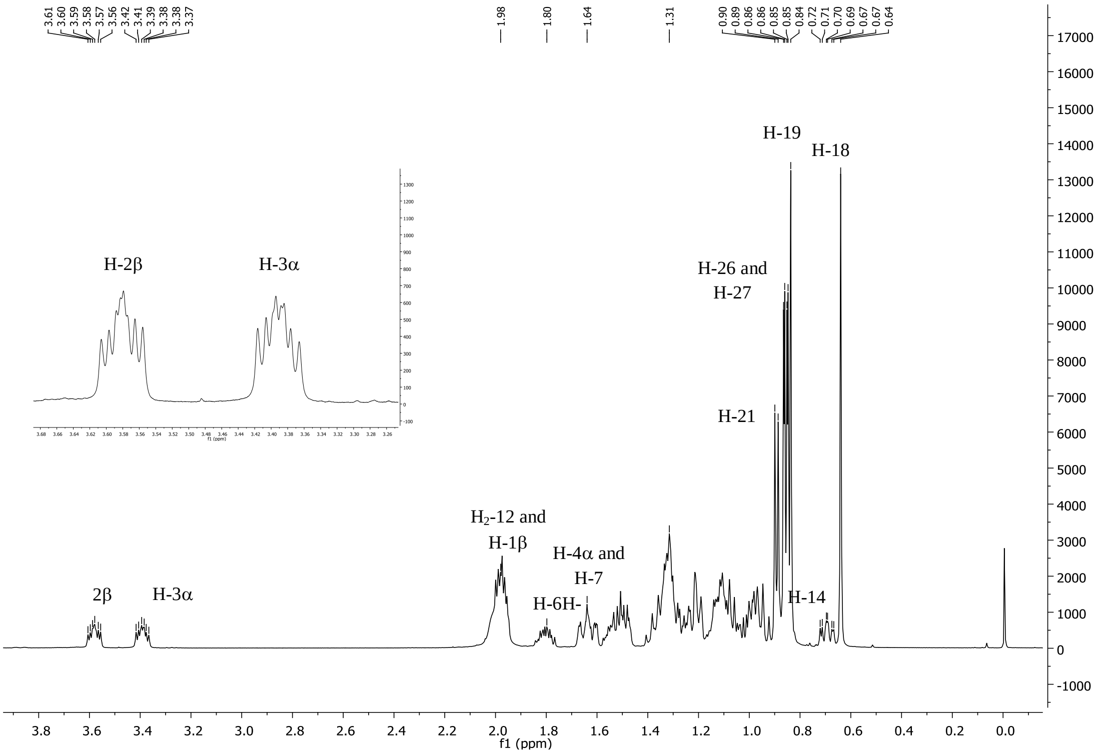

Figure S1: ^{1}H NMR ($CDCl_3$, 500 MHz) of steroid **2**. Bandwidth at half height (W ½) values measured in the ^{1}H NMR for H-2 and H-3 (27.5Hz and 26.4 Hz) are in accordance with a *trans* dihydroxyl system with both –OH at equatorial positions. This was confirmed by NOESY correlations.

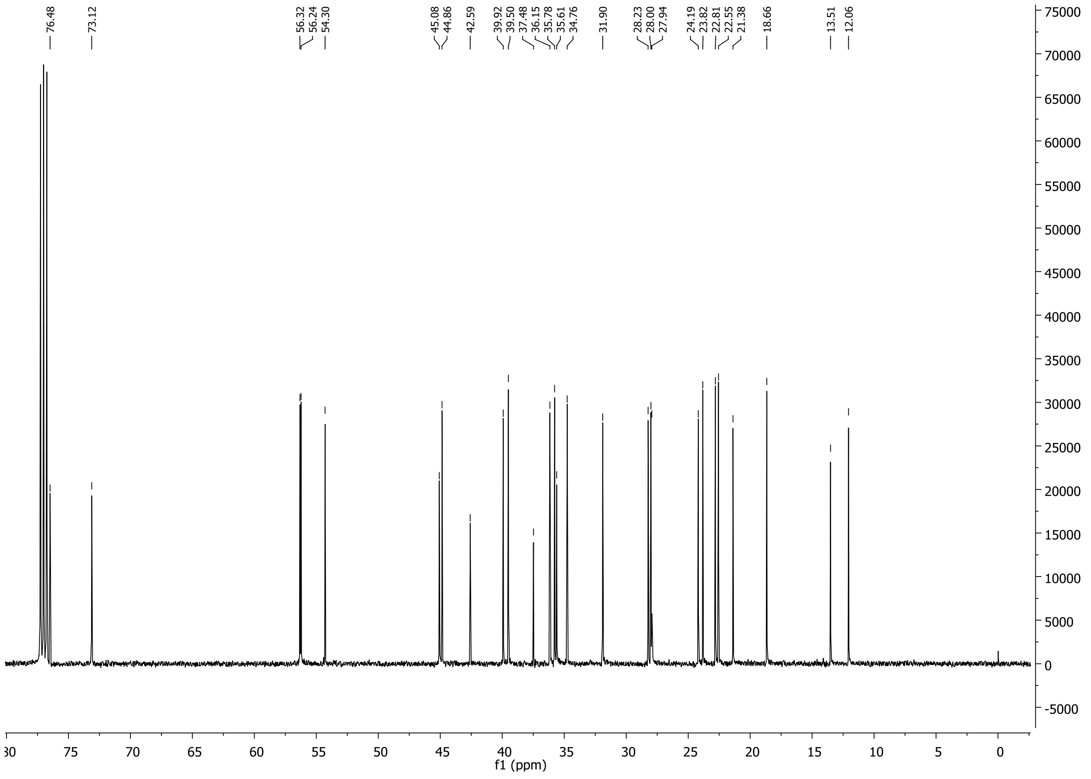

Figure S2: ^{13}C NMR ($CDCl_3$, 125 MHz) of steroid **2**.

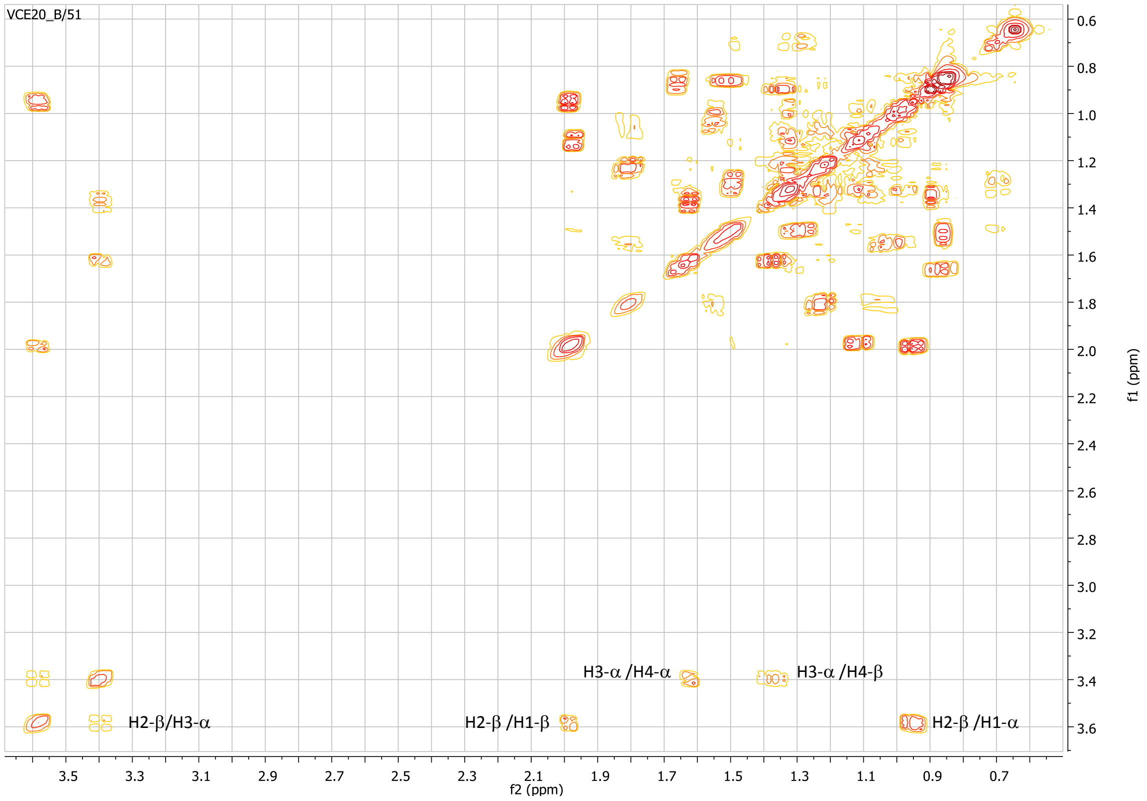

Figure S3: COSY ($CDCl_3$, 500 MHz) of steroid **2**.

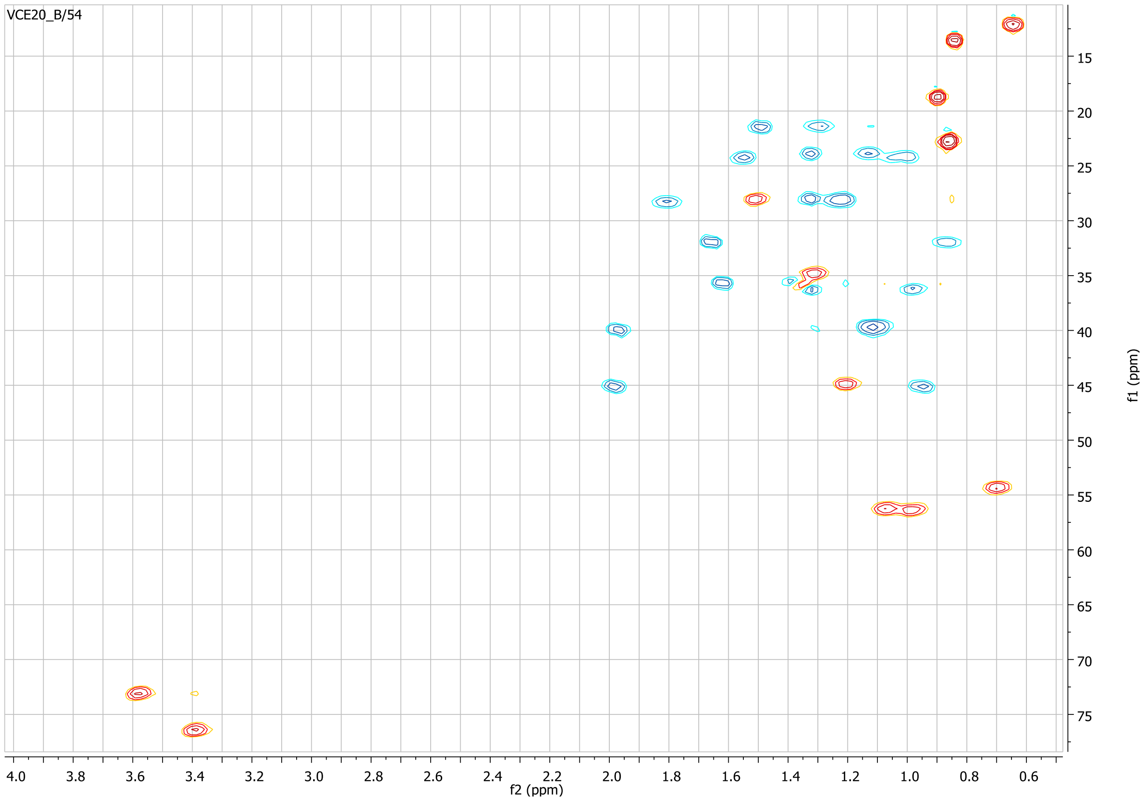

Figure S4: HSQC ($CDCl_3$, 500 MHz) of steroid **2**.

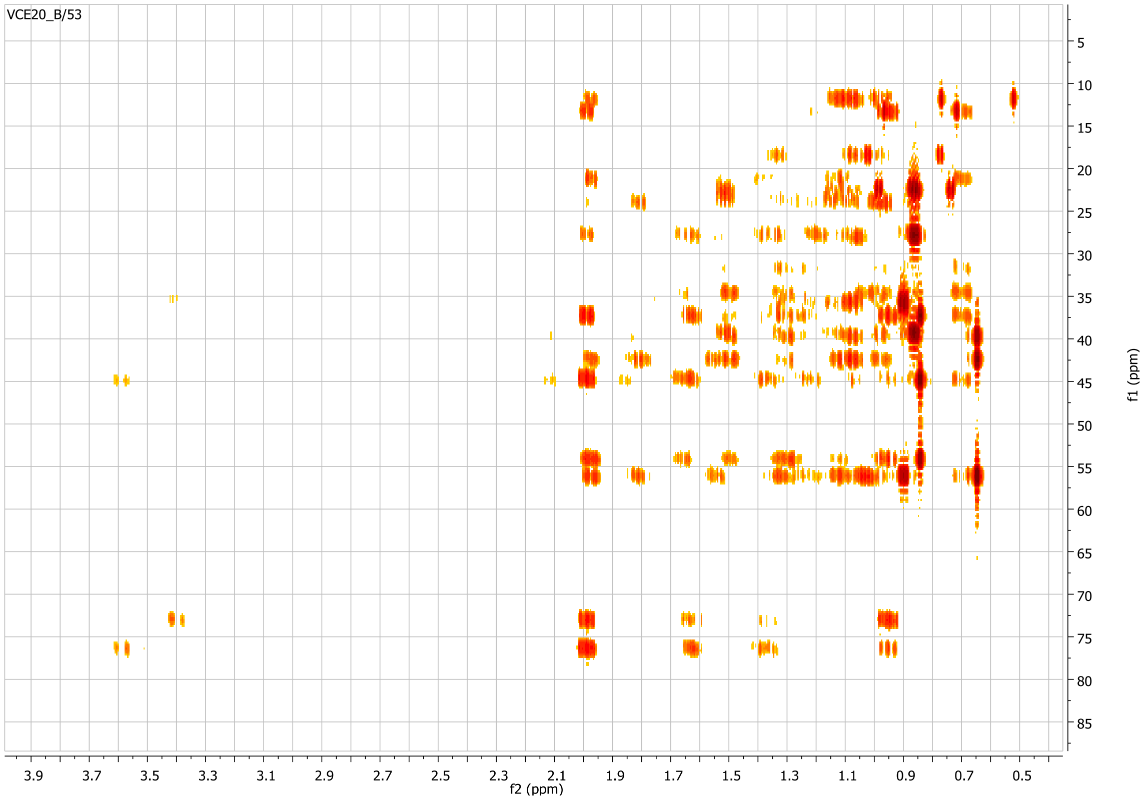

Figure S5: HMBC ($CDCl_3$, 500 MHz) of steroid **2**.

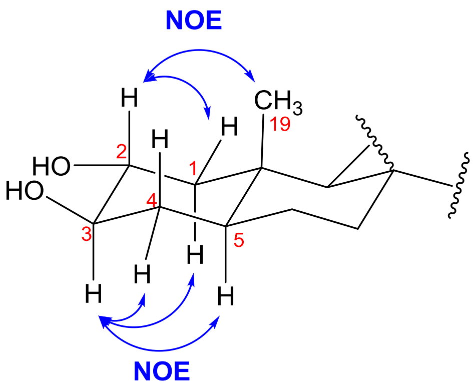

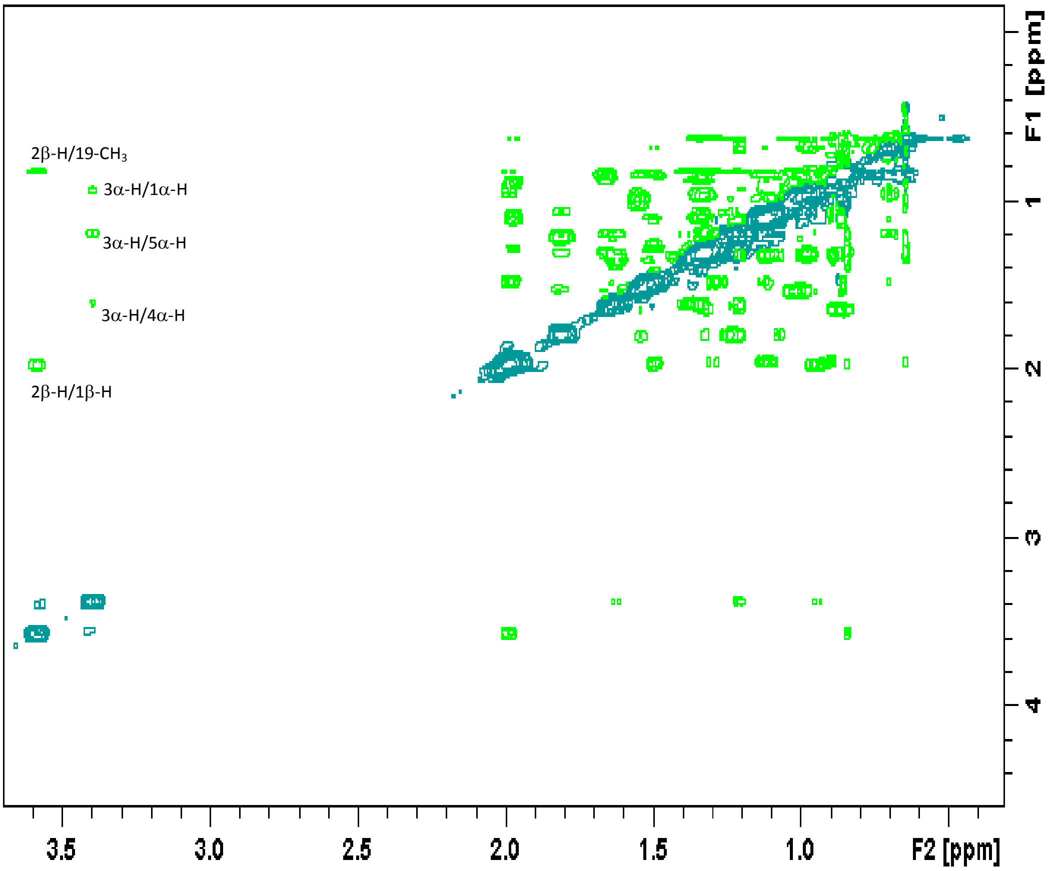

Figure S6: NOESY ($CDCl_3$, 500 MHz) of steroid **2.** The 2α-OH orientation was confirmed by a NOESY experiment were 2β-H showed a strong correlation with 19-CH_3 and 1β-H. NOE of 3-H were only observed with 1α, 3α and 4α.

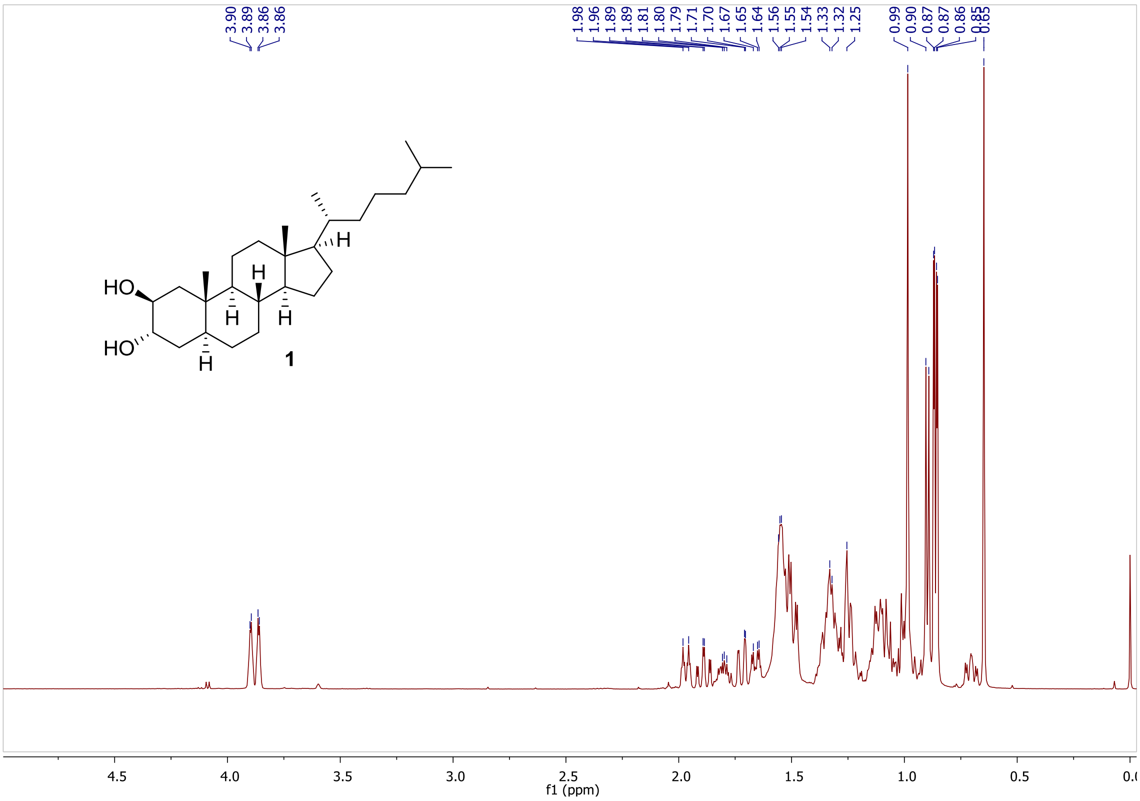

Figure S7: ^{1}H NMR ($CDCl_3$, 500 MHz) of steroid **1**.

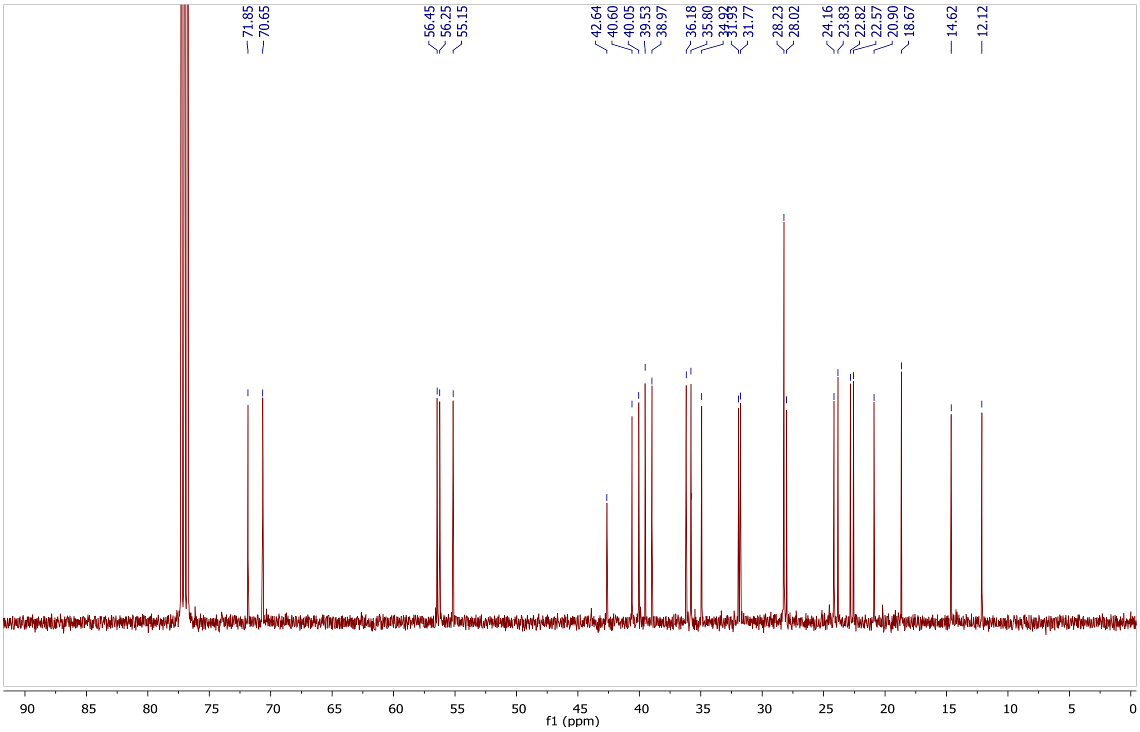

Figure S8: ^{13}C NMR ($CDCl_3$, 125 MHz) of steroid **1**.

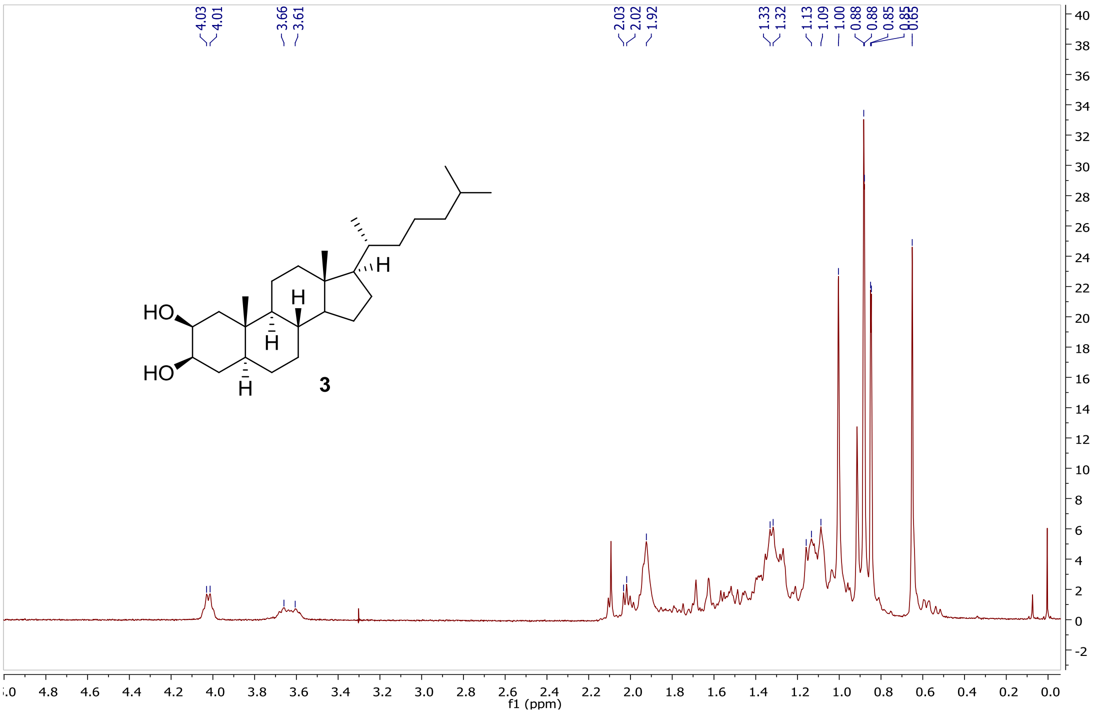

Figure S9: ^{1}H NMR ($CDCl_3$, 200 MHz) of steroid **3**.

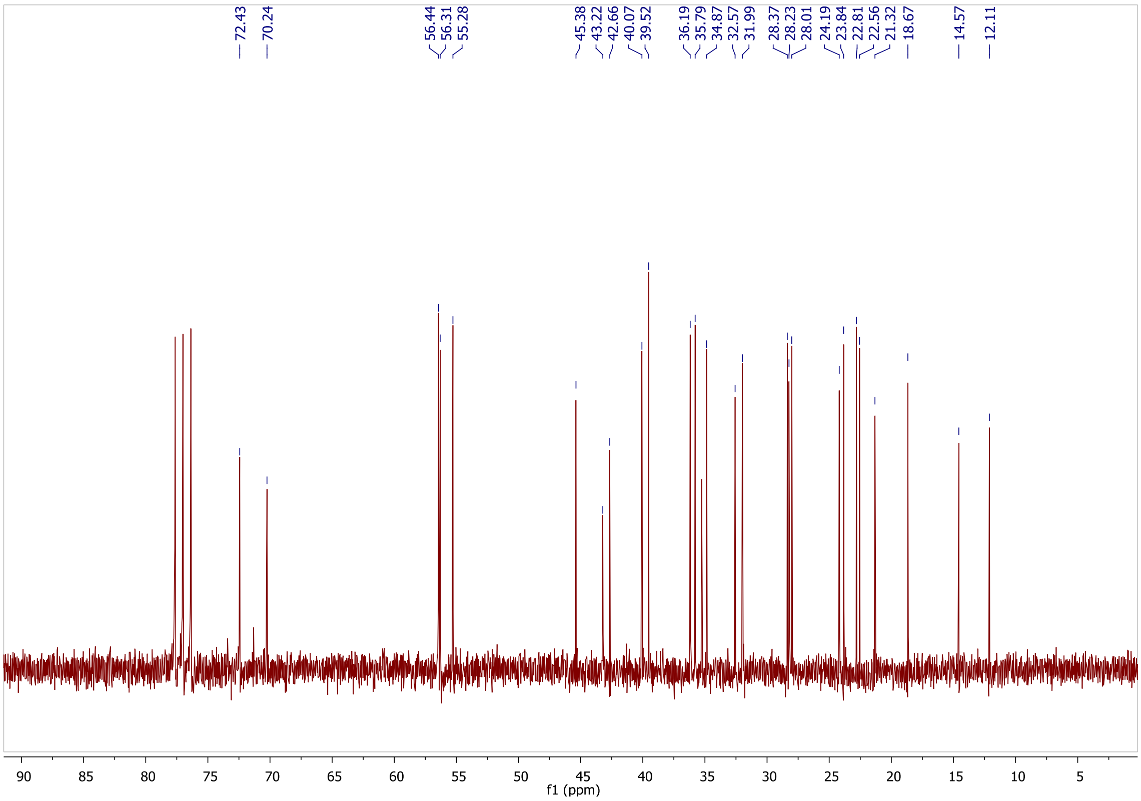

Figure S10: ^{13}C NMR ($CDCl_3$, 50 MHz) of steroid **3**.

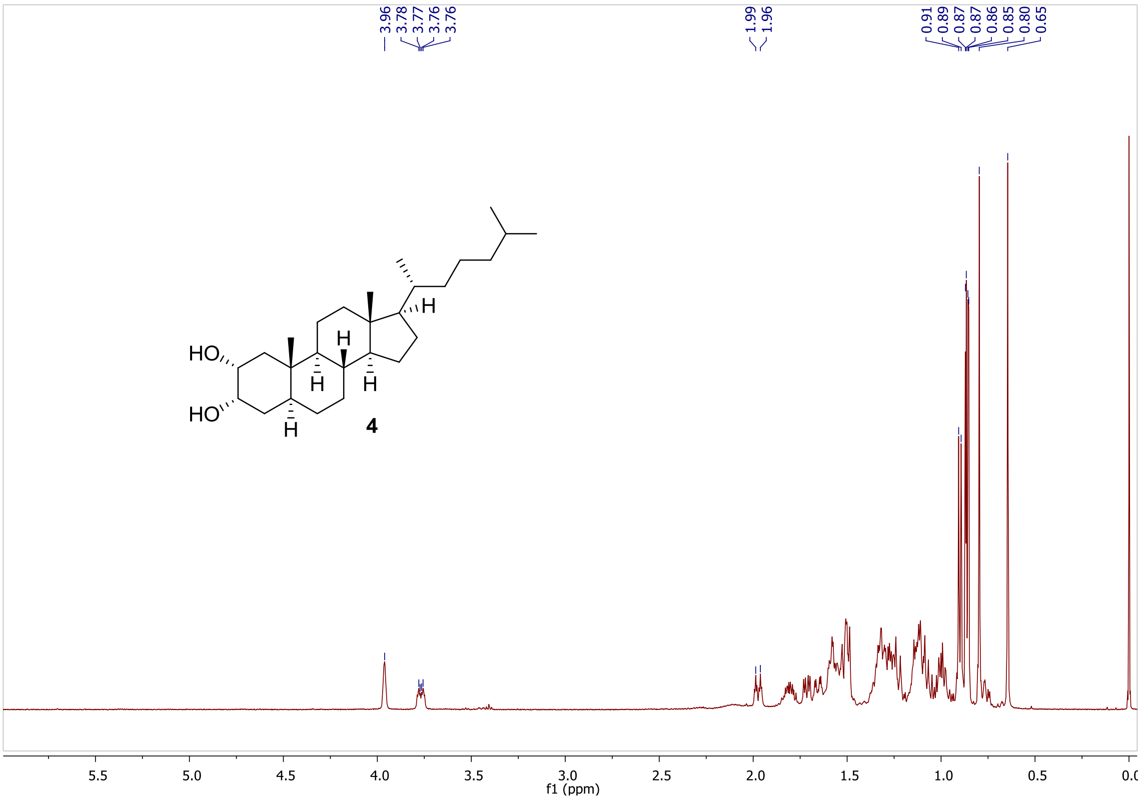

Figure S11: ^{1}H NMR ($CDCl_3$, 200 MHz) of steroid **4**.

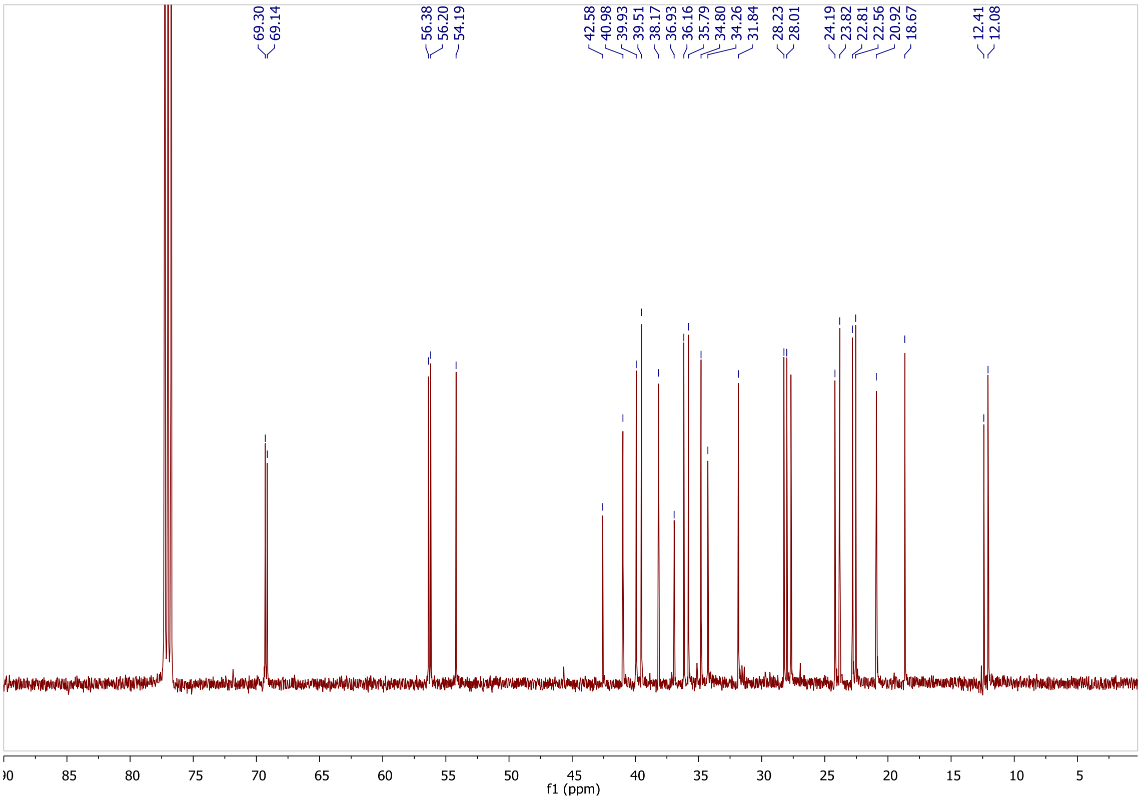

Figure S12: ^{13}C NMR ($CDCl_3$, 50 MHz) of steroid **4**.

Table S1: Hansen parameters of solvents used in this work.

	delta d	delta p	delta h	
n-Hexane	14.9	0	0	G
n-Decane	15.8	0	0	G
Methylcyclohexane	16.0	0	1.0	G
Cyclohexane	16.8	0	0.2	G
Carbon tetrachloride	17.8	0	0.6	G
Chloroform	17.8	3.1	5.7	G
Xylenes	17.8	1.0	3.1	G
Dichloromethane	18.2	6.3	6.1	G
Dimethyl sulfoxide	18.4	16.4	10.2	G
Styrene	18.6	1.0	4.1	G
1,2-Dichloroethane	19.0	7.36	4.09	G
Dioxane	19.0	1.8	7.4	G
n-Heptane	15.3	0	0	G
o-Dichlorobenzene	19.2	6.3	3.3	G
Aniline	19,4	5.1	10.2	G
Methyl acrylate	15.3	9.3	5.9	G
Toluene	18.0	1.4	2.0	G
Nitrobenzene	20.0	8.6	4.1	
Acetone	15.5	10.4	7.0	NG
Acetonitrile	15.3	18	6.1	NG
Dimethylformamide	17.4	13.7	11.3	NG
Ethanol	15.8	8.8	19.4	NG
Ethyl acetate	15.8	5.3	7.2	NG
Methanol	15.1	12.3	22.3	NG
n-Hexanol	15.9	5.8	12.5	NG
Tetraethoxysilane	13.9	4.3	0.6	NG
Tetrahydrofuran	16.8	5.7	8.0	NG
Triethylamine	14.9	5.74	5.99	NG
Water	15.5	16	42.4	NG
Pyridine	19.0	8.8	5.9	NG
Acetic acid	14.5	8	13.5	NG
n-Butanol	16.0	5.7	15.8	NG

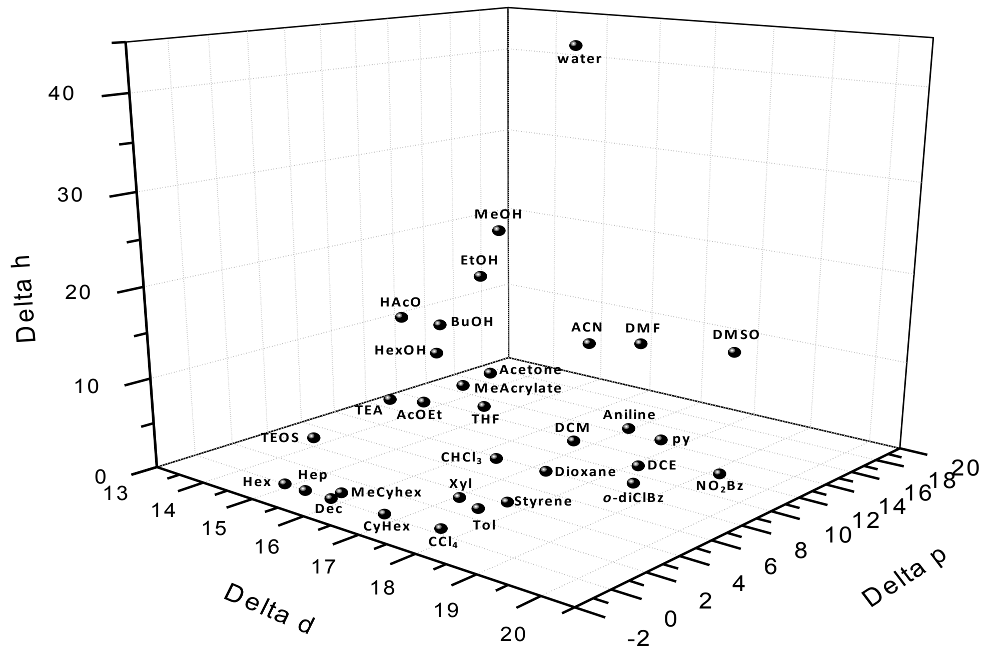

Figure S13: HSP 3D plot with explicit solvents.

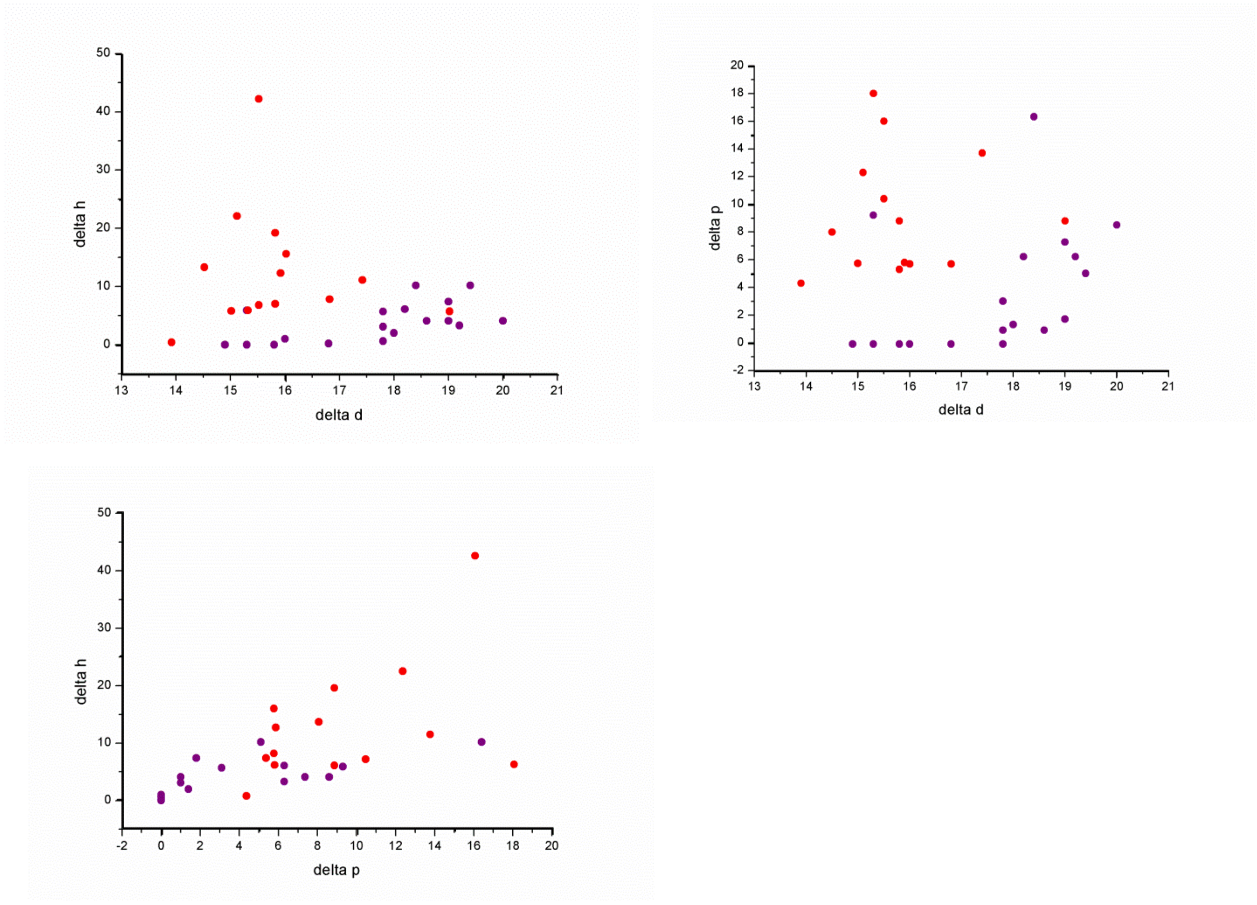

Figure S14: Bidimensional projections of the HSP plots. Purple: non gelated solvents, red: gelated solvents.

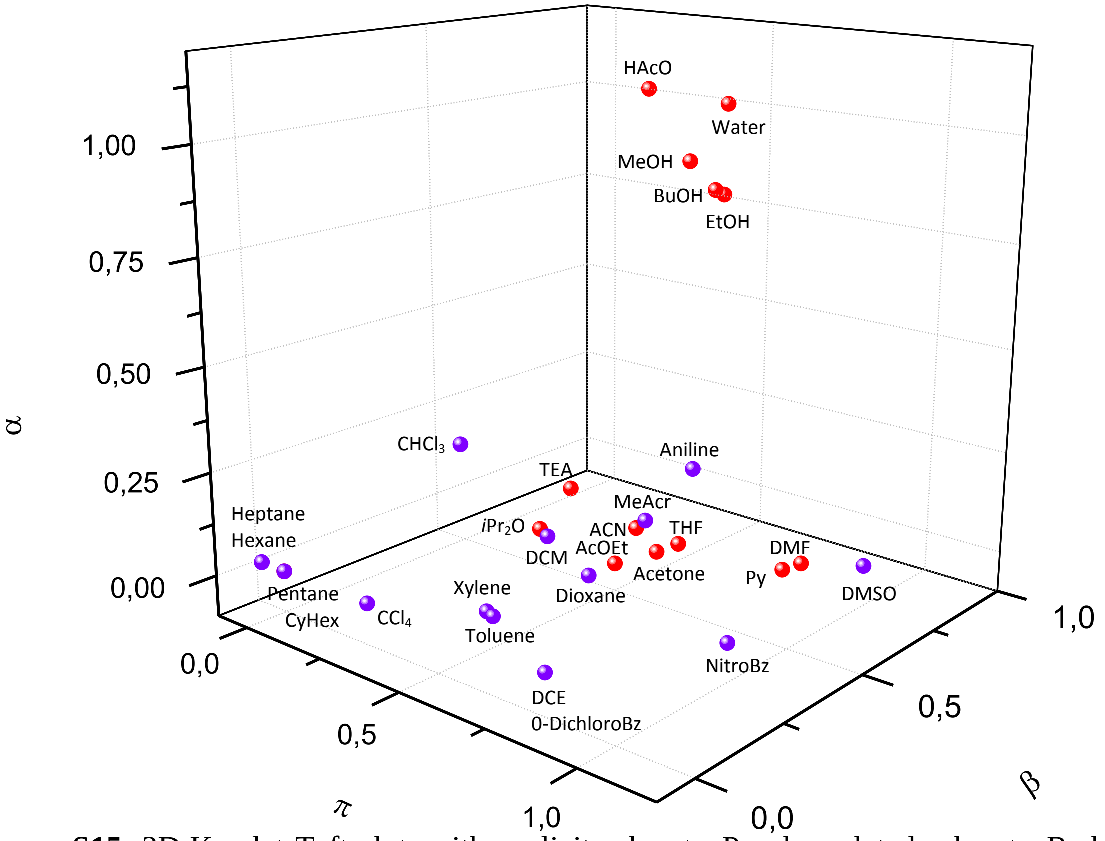

Figure S15: 3D Kamlet-Taft plots with explicit solvents. Purple: gelated solvents. Red: non gelated solvents.

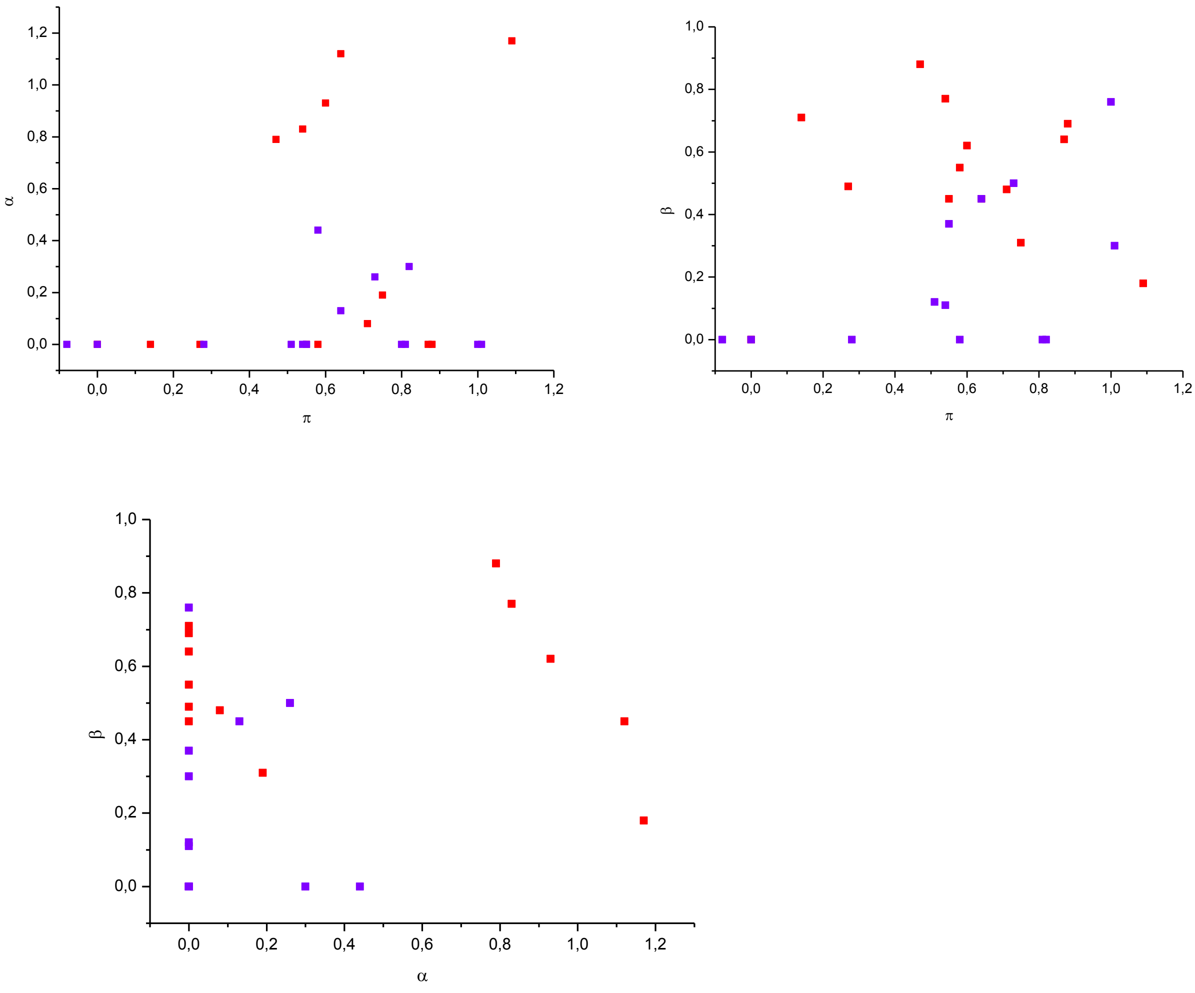

Figure S16: 1D Kamlet–Taft plots.

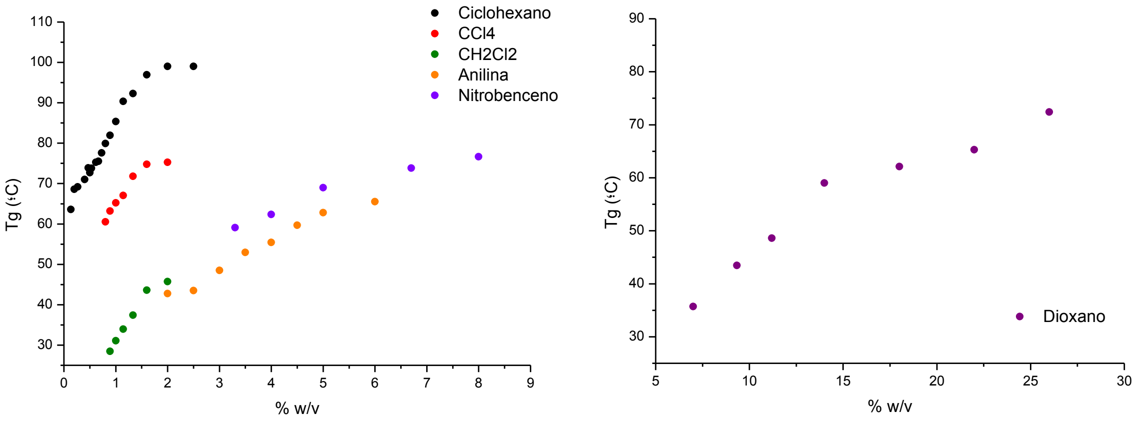

Figure S17: T_g-vs-concentration plots.

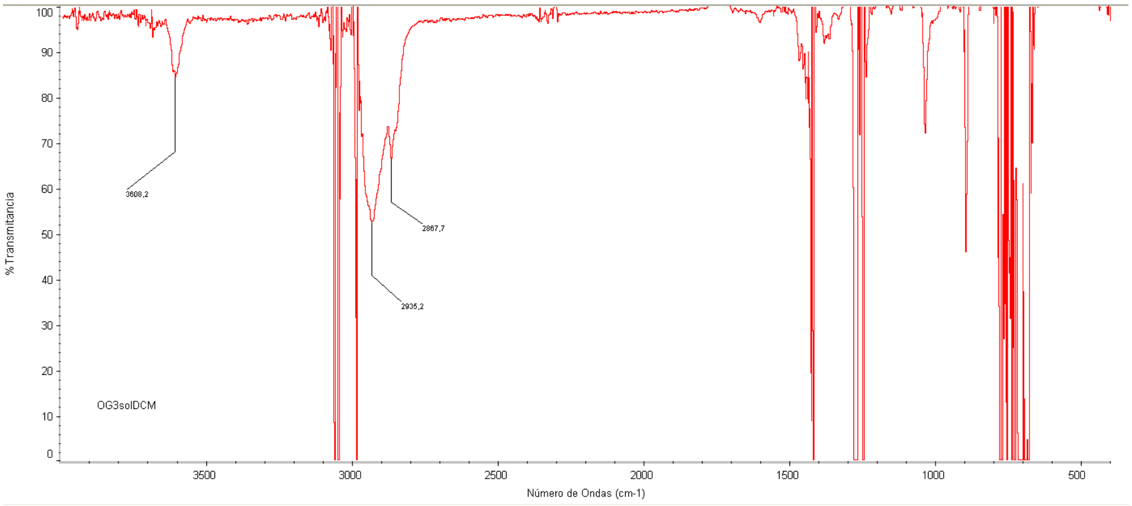

Figure S18: FTIR spectra for a solution of **1** in dichloromethane (concentration < CCG).

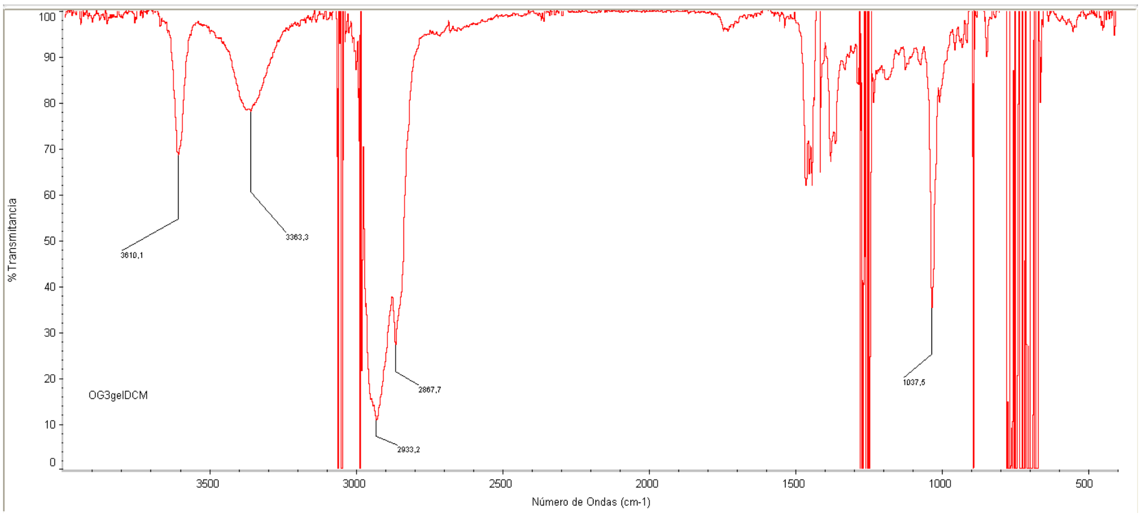

Figure S19: FTIR spectra for a gel of **1** in dichloromethane.

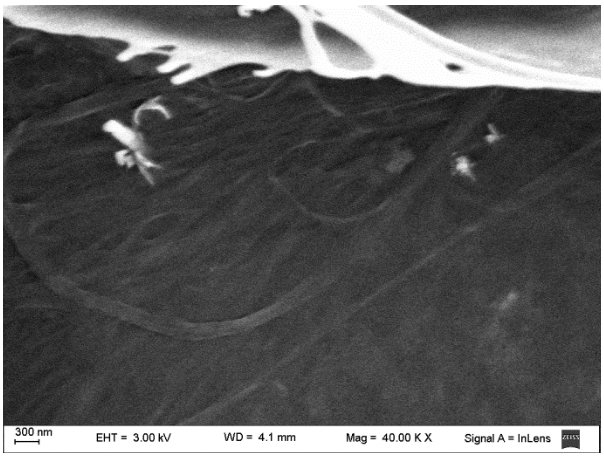

Figure S20: SEM image of the xerogel from *n*-hexane of LMOG **1**.

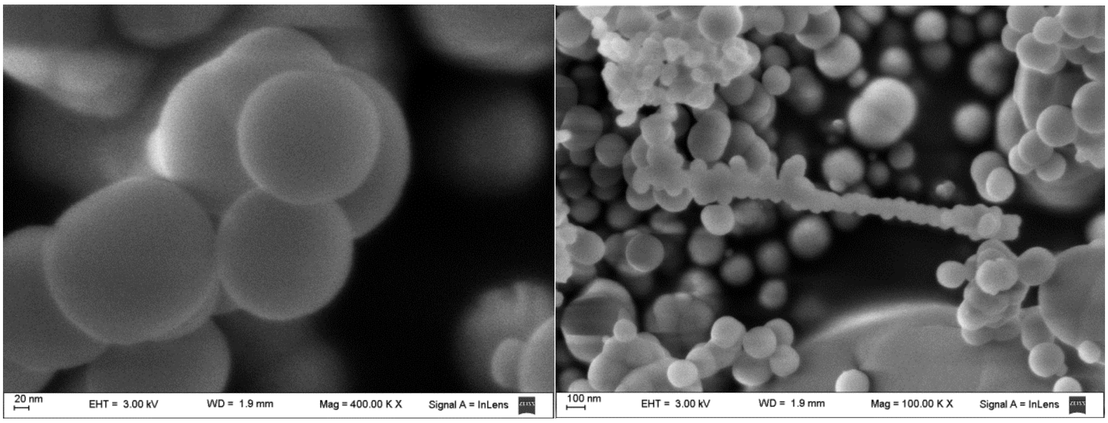

Figure S20: SEM images of silica nanoparticles obtained from in-situ polymerization of TEOS in dicloromethane gels of LMOG **1** (1 μL of benzylamine and 15μL of water).

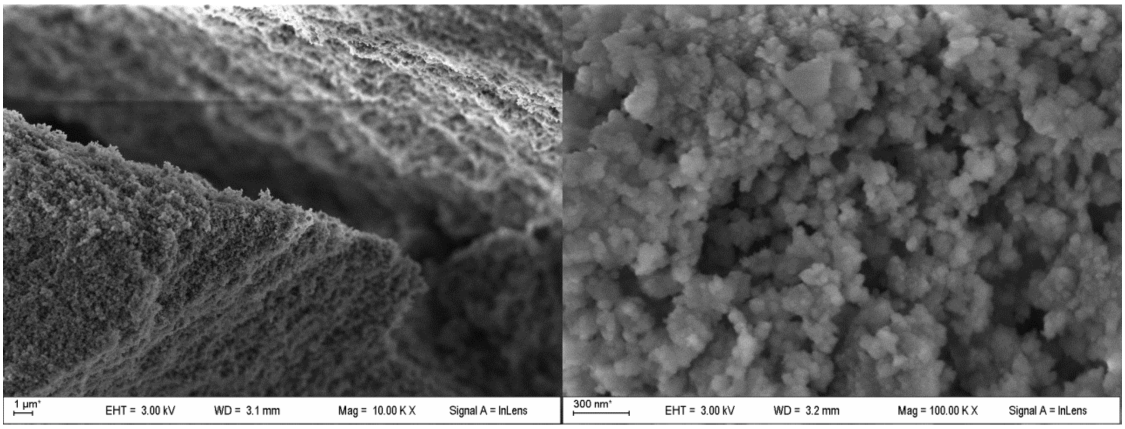

Figure S21: SEM images of amorphous silica nanoparticles obtained from in-situ polymerization of TEOS in dioxane solution (1 μL of benzylamine and 15 μL of water).
